# MVGAE: A Multi-View Graph Auto-Encoder Model for Drug Prediction of Non-Small Cell Lung Cancer Based on Synthetic Lethality

**DOI:** 10.3390/cimb48030269

**Published:** 2026-03-03

**Authors:** Shaobo Hu, Runsheng Jiang, Ning Zhao

**Affiliations:** College of Computer and Control Engineering, Northeast Forestry University, No. 26 Hexing Road, Harbin 150040, China; h1345519292@outlook.com (S.H.); 2023211621@nefu.edu.cn (R.J.)

**Keywords:** non-small cell lung cancer, synthetic lethality, drug repurposing, integrated computational framework

## Abstract

**Highlights:**

**What are the main findings?**

1. This study predicts nine key therapeutic target genes in non-small cell lung cancer (NSCLC) and identified their corresponding potential targeted drugs.

**What are the implications of the main findings?**

2. Provides experimentally testable target and drug candidates for NSCLC therapy.
3. Establishes an extensible computational pipeline for multi-omics target discovery.

**Abstract:**

Identifying therapeutic target genes and their corresponding targeted drugs is of significant importance for the treatment of non-small cell lung cancer (NSCLC). This study proposes a multi-view graph auto-encoder model (MVGAE), which, together with the network-informed adaptive positive-unlabeled (NIAPU) and synthetic lethality multi-view graph auto-encoder (SLMGAE) model, constitutes an integrated computational framework. The framework integrates multi-source biological network data, including protein–protein interaction networks, disease-gene association information, and gene-drug bipartite graphs, for data mining. Through systematic analysis and computational screening, we ultimately predicted seven potential driver genes associated with NSCLC using the NIAPU model. The SLMGAE model predicted nine genes with synthetic lethality (SL) interactions to these driver genes as candidate therapeutic targets. Based on these SL targets, the MVGAE model further predicted corresponding targeted drugs. Notably, among the prioritized targets, existing studies indicate that *ATR* and *RAD51* exhibit conditional SL effects in the context of functional impairment. Furthermore, several of the predicted candidate drugs (such as PAZOPANIB) have been previously reported to play a positive role in NSCLC treatment. This study highlights MVGAE as a novel computational framework for drug repurposing and demonstrates how its integration with complementary models can effectively prioritize potential therapeutic targets and candidate drugs, providing a robust computational basis for precision treatment strategies.

## 1. Introduction

Lung cancer remains the leading cause of cancer-related deaths worldwide. In 2022, it was the most commonly diagnosed cancer, with nearly 2.5 million new cases, accounting for 12.4% of all cancers globally [1]. Among these, non-small cell lung cancer (NSCLC) constitutes over 85% of cases, representing the predominant subtype of lung cancer [2].

Exploring gene-disease associations is crucial for understanding the underlying mechanisms of disease pathogenesis, holding significant implications for disease prevention and treatment strategies. Therefore, identifying pathogenic genes is essential for the effective treatment of NSCLC. However, a major challenge in the computational identification of disease genes lies in the ambiguous background caused by unknown or truly unrelated genes. To date, traditional methods based on gene expression, genome-wide association studies (GWAS), or clinical trials have proven effective in discovering disease-associated genes. However, they remain highly time-consuming and costly [3,4,5,6]. Meanwhile, with the rapid advancement of DNA sequencing technology, an increasing number of biological databases have been established, providing abundant data for the study of pathogenic genes. Therefore, to address these issues, researchers have begun to develop database-driven computational approaches to predict genes associated with diseases. In the field of machine learning (ML), this task is formulated as identifying new positive instances from a set consisting of positive and unlabeled samples, which is known as Positive–Unlabeled (PU) learning [7]. For example, Yang et al. [8] proposed a two-step technique that introduces a multi-class labeling scheme consisting of five different labels, namely Positive (P), Likely Positive (LP), Weak Negative (WN), Likely Negative (LN), and Reliable Negative (RN). The goal of this method is to relabel instances and subsequently train a supervised learning algorithm. On this basis, Paola Stolfi et al. [9] further improved the multi-class approach and proposed the NIAPU model, which incorporates more reasonable constraints and specific network-based features in both the distance-matrix construction and class partitioning processes, enabling the method to be applied to more general PU learning tasks.

**Figure 1:**
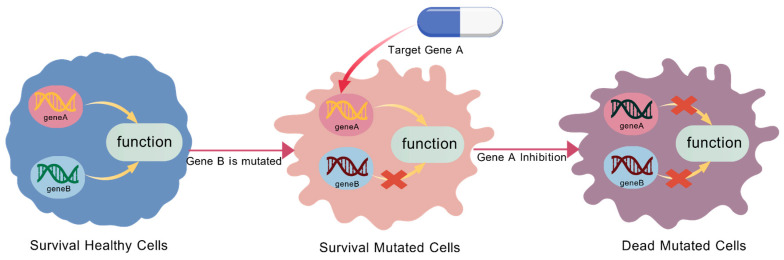
Selective killing of cancer cells via the Synthetic lethality (SL) mechanism [20].

Despite substantial advances in targeted therapy and immunotherapy, the treatment of NSCLC still faces major challenges. Taking targeted therapy as an example, tumor cells can develop drug resistance through multiple mechanisms, including target modification and activation of alternative signaling pathways. The former refers to the acquisition of new mutations in cancer cells, which leads to resistance to specific therapies; the latter bypasses the original pathway and activates cell survival and proliferation signaling, a process known as “adaptive resistance” [10]. Due to the high degree of genetic and molecular heterogeneity in NSCLC, its driver targets are prone to diverse modifying mutations and exhibit pronounced target-modification characteristics. As a result, targeted therapies are often effective only against dominant tumor clones, but fail to eradicate subclones harboring resistance-associated mutations [11]. Meanwhile, although immunotherapy has shown great potential in NSCLC, a subset of patients still develops resistance or exhibits limited clinical benefit [12]. These effects may be attributed to several underlying mechanisms, including the loss of immune recognition, immunosuppressive remodeling of the tumor microenvironment, dysregulated activation of immune checkpoints, and substantial intratumoral heterogeneity [13,14,15,16,17]. These factors collectively hinder the long-term effectiveness of current therapeutic strategies for NSCLC. To address these challenges, researchers have proposed the concept of synthetic lethality (SL) for cancer therapy, which has attracted considerable attention in the field of oncology [18]. Specifically, SL refers to a genetic relationship in which the defect of a single gene is non-lethal, whereas the simultaneous inactivation of two genes results in cell death. When such a pair of genes exhibits this dependency, they are defined as an SL gene pair, and each gene is considered the SL partner of the other. In cancer cells harboring a mutation in one gene of an SL pair, therapeutic inhibition of its SL partner can selectively eliminate cancer cells while sparing normal cells, thereby achieving targeted cytotoxicity with minimal off-target toxicity [19]. The detailed mechanism is illustrated in Figure 1.

In contrast to traditional chemotherapy, SL-based therapeutic strategies exhibit reduced toxicity and fewer side effects [21]. This concept has been successfully translated into clinical applications; for instance, the SL interaction between PARP and BRCA1/BRCA2 has been employed in the clinical treatment of ovarian and breast cancers [22]. To further advance the application of this concept in cancer therapy, researchers have conducted wet-lab experiments and employed CRISPR-based genome editing technologies to identify SL interactions [23,24]. However, both of these approaches require substantial time and financial investment when identifying SL gene pairs, and their success rates are often limited by off-target effects. Therefore, as an effective complement and guidance for experimental studies, a number of machine learning–based methods have been proposed to predict SL gene pairs. In particular, graph neural networks (GNNs) have been widely applied in various network-related tasks owing to their strong capability in modeling complex graph-structured data. Among these models, the graph convolutional network (GCN) is one of the most widely adopted for its ability in learning from irregular graph-structured data and aggregating information from neighboring nodes to capture global structural patterns. By modeling genes as nodes and SL relationships as edges, GCNs leverage neighborhood aggregation mechanisms to learn informative node representations while simultaneously characterizing the potential interaction patterns between genes. For example, Zhu et al. proposed SLGNN, a knowledge graph-based graph neural network model that models gene preferences under different relational contexts within a knowledge graph [25]. In addition, Hao et al. developed SLMGAE, a multi-view graph auto-encoder model that uses the SL network as the primary view and incorporates protein–protein interaction (PPI) networks and Gene Ontology (GO) annotations as supporting views. The model reconstructs these views through a graph auto-encoder (GAE) framework to predict SL interactions, and has demonstrated superior predictive performance, providing crucial methodological foundations for this study [26].

Parallel to the challenges of target identification, the success rate of traditional drug development is extremely low, with only about 11% of candidates approved in Phase I clinical trials. Moreover, the development of a new drug typically requires an investment of 2–3 billion USD and takes approximately 10–17 years to complete. In contrast, drug repurposing substantially reduces development risk and cost, enabling drugs to reach the market within 3–12 years with an average investment of only about 300 million USD [27]. Therefore, in order to reduce the substantial time and financial costs associated with de novo drug development, increasing attention has been directed toward drug repurposing, which aims to rediscover and exploit existing approved drugs for novel therapeutic indications. Prominent historical successes underscore the viability of this strategy. For instance, thalidomide was originally introduced in the 1950s as a sedative and antiemetic agent for pregnant women; however, subsequent studies revealed its therapeutic efficacy, and it has since become the only approved treatment for erythema nodosum leprosum [28]. Compared with traditional drug repurposing strategies that primarily rely on clinical discovery, computational approaches are able to generate predictions in a more rational and cost-effective manner, thereby substantially narrowing the candidate space for experimental validation [27]. For example, Gottlieb et al. identified previously unknown drug–disease associations by integrating drug similarity and disease similarity information [29]. Palhamkhani et al. proposed DeepCompoundNet, a deep learning-based model that integrates protein features, drug properties, and multiple interaction data sources to predict chemical–protein interactions [30]. Xuan et al. introduced CNNMDA, a convolutional neural network-based framework for predicting disease-associated miRNAs, which combines network representation learning with CNN architectures [31]. In addition, Fan et al. developed SGCLDGA, a model that employs graph neural networks together with self-supervised contrastive learning to predict unknown drug–gene associations. This model applies singular value decomposition (SVD) and performs contrastive learning across multiple views, thereby enhancing the quality of vector representations learned by GCNs for drugs and genes [32].

Despite substantial progress in each of these individual domains, there remains a critical need for a unified computational framework that can systematically cover the three key stages of disease-related gene identification, SL gene pair prediction, and drug repurposing. Most existing methods are usually designed to address only a single task—for example, focusing exclusively on disease gene identification, SL interaction prediction, or drug repurposing—and therefore fail to reveal the complete biological chain from disease pathogenesis, to therapeutic target discovery, and finally to candidate drug screening. In the absence of such an integrated computational paradigm, these methods, despite their effectiveness within specific problem domains, are difficult to directly translate into disease-oriented research and clinical applications due to the lack of a unified dataset and analysis workflow.

To address this gap, in this study we integrate multiple heterogeneous biomedical data sources, including gene–disease associations (GDA), SL gene interaction networks, drug–target relationships, GO annotations, and PPI networks. We demonstrate the clinical utility of this framework using NSCLC as a representative case study. The framework organically combines the NIAPU and SLMGAE models to construct a complete analytical pipeline, ranging from the identification of potential NSCLC-associated driver genes, to the screening of synergistic therapeutic targets based on the SL concept, and finally to drug repurposing guided by the identified gene targets.

In particular, this study further extends the application scope of SLMGAE from SL interaction prediction to drug–gene association prediction. To accommodate this new application, the model was adapted and redefined as the Multi-view Graph Auto-Encoder (MVGAE). By integrating multi-view information from both drugs and genes, MVGAE overcomes the limitations of relying on a single drug–gene interaction network, enabling a more comprehensive exploration of potential drug–gene relationships.

**Figure 2:**
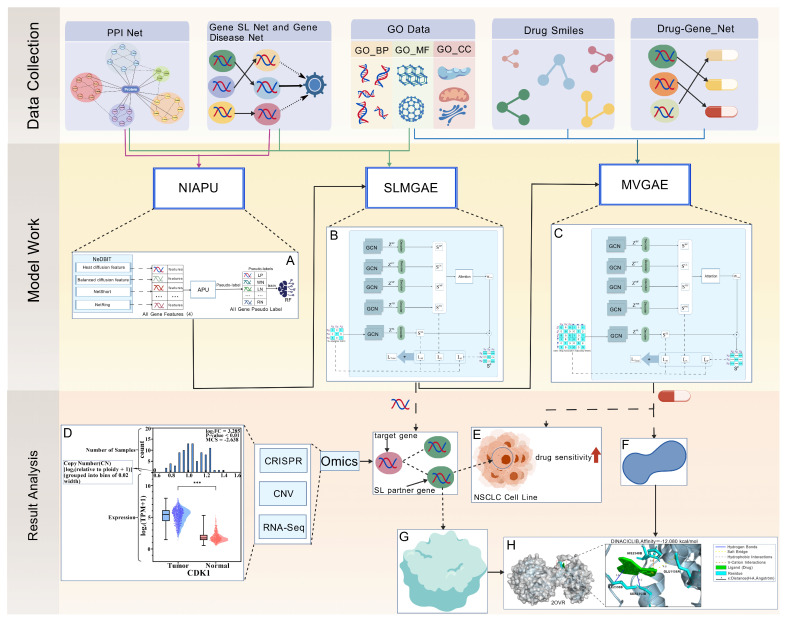
Overview of the research workflow. (1) Data collection: Multi-omics datasets required for this study were obtained from publicly available databases (see Section 2.1 for detailed data sources). (2) Model workflow: The data were processed sequentially by three computational models, where the output of each model served as the input to the next one ((**A**–**C**) correspond to the architectures of the three models, as detailed in Section 2.2, Section 2.3 and Section 2.4). (3) Result analysis: The integrated computational framework was used to analyze and filter the outputs, enabling the identification of potential pathogenic driver genes in NSCLC and their associated Synthetic lethality (SL) partner genes, as well as the candidate drugs targeting these SL genes. (4) Case study using CDK1 as an example: (**D**) shows the multi-omics characteristics of CDK1 in NSCLC, including: (i) RNA-Seq expression differences between NSCLC cell lines and normal controls; (ii) distribution of DNA copy number (CN) across NSCLC cell lines (where “count” denotes the number of cell lines corresponding to each CN with a bin width of 0.05); (iii) mean Chronos Score (MCS) in NSCLC-related cell lines. (5) Candidate drugs were prioritized based on their increased sensitivity in NSCLC cell lines (**E**). (6) Molecular docking validation: The PDB-encoded protein structure of the target gene (**G**) and the molecular structure of the candidate drug (**F**) were retrieved, and molecular docking was performed to evaluate their binding potential. Panel (**H**) shows an example of docking between the FBXW7 protein (PDB: 2OVR) and the drug DINACICLIB [20].

Through this unified computational framework, we not only integrate multi-source biological knowledge and multiple models within a coherent theoretical paradigm, enabling a full-chain inference process from genes to drugs, but also provide prioritized candidate genes and drug combinations with higher practical value for subsequent experimental validation and potential clinical translation, thereby offering new insights and powerful tools for precision therapy in NSCLC. Our research workflow is shown in Figure 2.

## 2. Materials and Methods

### 2.1. Data Source

This study integrated multi-source data from several public databases. Gene data associated with NSCLC were obtained from the Comparative Toxicogenomics Database (CTD, accessed on 19 September 2025) [33,34]. In total, CTD contains 23,206 genes linked to NSCLC, supported by three types of evidence: clinically validated data, literature curation, and computational inference. Among them, 16,930 genes belong to the human gene set; however, 16,786 of these genes are mainly derived from literature reports or computational prediction approaches, and therefore lack rigorous clinical validation and remain uncertain to some extent. To enhance the reliability of downstream analyses, we further extracted 144 genes from the above set that were annotated in CTD as “Marker/Mechanism” or “Therapeutic”. These genes are supported by clinical evidence and have been confirmed as NSCLC-related biomarkers, key mechanistic contributors, or therapeutic targets, and were thus retained as a high-confidence gene set in this study. In addition, we downloaded the latest version of the human PPI data from BioGRID 4.4 [35,36] (accessed on 11 September 2025). After filtering and preprocessing, non-human genes and genes lacking GO annotations (GO_CC, GO_BP, or GO_MF) were removed, along with their corresponding interaction edges. A total of 878,761 interaction edges involving 18,678 gene nodes were retained. This PPI network was used in this study as a global background interaction network for graph construction and feature propagation; furthermore, we collected SL interaction data from SynLethDB 3.0 [37] (accessed on 11 September 2025), which is one of the most comprehensive and up-to-date databases of human SL interactions. Restricting to edges where both genes are present in the aforementioned set of 18,678 genes, a total of 22,966 SL interaction edges were retained, each supported by functional experimental validation, or high-throughput screening data in the database. These SL interactions were used as a reliable benchmark dataset for SL network construction and model training in this study. In addition, the latest gene–drug interaction data were retrieved from DGIDB v5.0.10 [38,39] (accessed on 10 September 2025). After removing edges involving non-human genes or those with unrecorded association types (e.g., activation, inhibition), a total of 1734 confirmed gene–drug interaction edges involving 1263 drug entities were retained. These interaction data served as an essential foundational resource for the subsequent analyses in our computational framework. Gene identifiers from different databases were standardized using Entrez Gene IDs. Overall, the sources and statistical summaries of all datasets used in this study are presented in Table 1.

### 2.2. Predict Potential Driver Genes in NSCLC

This module aims to predict potential disease-associated genes in NSCLC. Among the 18,678 genes, only 144 genes are labeled as positive genes P (referred to as seed genes), while the remaining 18,534 genes constitute the unlabeled set U, forming a typical *PU* task. Therefore, we adopt the NIAPU model proposed by Paola Stolfi et al. [9]. This model is an improved *PU* learning framework, which, based on Yang et al. [8], introduces and refines a multi-class labeling approach aimed at identifying the *LP* set within the unlabeled set U that is similar in features to the positive set P. Genes in this LP set share characteristics with those in P and are therefore prioritized as candidate disease genes. The fundamental idea is based on the separability and smoothness assumptions: positive examples P should be distinguishable from unlabeled samples U in the feature space, and instances with similar features are more likely to share the same label. NIAPU implements this principle through a two-stage procedure. The first stage is feature engineering: this stage introduces features based on network diffusion and biological information topology (NeDBIT), specifically including heat diffusion features and balanced diffusion, as well as two biological topological metrics, NetShort and NetRing.

The heat diffusion features are obtained by implementing a heat diffusion process on the gene PPI network. On the network, the evolution of the weights over time is described by the diffusion equation on graphs [40]:

(1) $$\begin{array}{c} z^{\prime }\left(t\right)+Lz\left(t\right)=0, \end{array}$$

Here, L denotes the graph Laplacian matrix, which is defined based on the adjacency matrix A of the PPI network and the degrees, with a diagonal matrix K satisfying $K_{ii}=k_{i}$:

(2) $$\begin{array}{c} L=K-A. \end{array}$$

The analytical solution of the above differential equation provides the distribution of node weights at any time t:

(3) $$\begin{array}{c} z\left(t\right)=exp\left(-Lt\right)z\left(0\right), \end{array}$$

where exp(⋅) denotes the matrix exponential. The initial weights are set as:

(4) $$\begin{array}{c} z_{i}\left(0\right)=\left\{\begin{array}{cc} s_{i}, & i\in \Sigma \\ 0, & i\notin \Sigma \end{array}\right., \end{array}$$

where $s_{i}$ is the disease association score of the gene, and Σ denotes the set of seed genes.

The balanced diffusion features are also based on the diffusion Equation (1), but use an alternative form of the graph Laplacian matrix:

(5) $$\begin{array}{c} L_{b}=I-K^{-1}A, \end{array}$$

That is, $L_{b}$ is used instead of the standard Laplacian matrix L. Consequently, the distribution of node weights at any time t is given by:

(6) $$\begin{array}{c} z\left(t\right)=exp(-L_{b}t)z\left(0\right), \end{array}$$

The initial weights are set in the same manner as in Equation (4).

The NetShort measure [41] evaluates the importance of a node based on its network distance to the seed genes. For each neighbor j of node i, the edge weight $w_{ij}$ is defined as:

(7) $$\begin{array}{c} w_{ij}=a_{ij}^{2}\left({\tilde{s}}_{i}+{\tilde{s}}_{j}\right), \end{array}$$

where $a_{ij}$ is an element of the PPI adjacency matrix A, and ${\tilde{s}}_{i}$ is the normalized association score of node i, defined as:

(8) $$\begin{array}{c} w_{ij}=a_{ij}\frac{2}{{\tilde{s}}_{i}+{\tilde{s}}_{j}},{\tilde{s}}_{i}=\left\{\begin{array}{cc} \frac{s_{i}}{maxS} & i\in \Sigma \\ \alpha \frac{minS}{maxS} & i\notin \Sigma \;\;\; \end{array}\right., \end{array}$$

Here, min S and max S denote the minimum and maximum values in the set of association scores, respectively, and α is the penalty parameter for non-seed nodes, typically set to 0.5 [8]. Then, the NetShort measure of node i, $NS_{i}$, is defined as:

(9) $$\begin{array}{c} {NS}_{i}=\sum_{j\neq i}\frac{1}{d_{ij}}, \end{array}$$

where $d_{ij}$ denotes the length of the weighted shortest path from node i to node j.

Subsequently, the NetRing measure is introduced based on the concept of ring structures. Given a set of seed genes, the network is partitioned into rings, each containing a group of nodes at the same distance from the seed nodes. Define:

(10) $$\begin{array}{c} R\left(l\right)\equiv \left\{j\in V|\underset{i\in \Sigma }{min}\;l_{ij}=l\right\}, \end{array}$$

where $l_{ij}$ is the length of the shortest unweighted path between nodes i and j. R(0) denotes the 0th ring containing all seed genes, and R(x),x=1,2,3,…,l, denotes the ring consisting of all nodes at distance x from the seed genes. The rings satisfy the following properties:

(11) $$\begin{array}{c} R\left(l_{1}\right)\cap R\left(l_{2}\right)=\emptyset \left(l_{1}\neq l_{2}\right), \end{array}$$

(12) $$\begin{array}{c} V=\overset{L}{\underset{l=0}{⋃}}R\left(l\right). \end{array}$$

The initial score of each node i is set as:

(13) $$\begin{array}{c} {\hat{r}}_{i}=\left\{\begin{array}{cc} 1-\frac{s_{i}}{maxS}, & i\in \Sigma \\ 1\;, & i\notin \Sigma \end{array}\right.. \end{array}$$

Then, the NetRing measure of node i, $r_{i}$, is defined as:

(14) $$\begin{array}{c} r_{i}=\left\{\begin{array}{ll} \alpha {\hat{r}}_{i}+(1-\alpha )\frac{1}{k_{i}}\sum_{j|A_{ij}\neq 0}\;{\hat{r}}_{j}\;\;\; & i\in \Sigma \\ l_{i}+\frac{1}{k_{i}}\left(\sum_{j\in O_{i}}\;{\hat{r}}_{j}+\sum_{j\in R_{i}\left(l_{i}-1\right)}\;r_{j}-\left(l_{i}-1\right)\right) & i\notin \Sigma \;\;\;\; \end{array},\right. \end{array}$$

According to the formula, the score of a seed gene is a convex combination of its initial ranking ${\hat{r}}_{i}$ and the average of the initial rankings of its neighbors, so that seed genes with many seed neighbors receive higher ranks. The ranking of non-seed nodes is calculated as the average of two terms: the number of genes in the same or higher-level ring $O_{i}=j\notin R\left(l-1\right)|A_{ij}\neq 0$, and the sum of the rankings of genes in lower rings modified by the ring level, $R_{i}\left(l_{i}-1\right)=j\in R\left(l_{i}-1\right)|A_{ij}\neq 0$.

Based on the above steps, the NeDBIT features for the genes were computed. Subsequently, the APU algorithm was applied to automatically classify and prioritize the unlabeled set U using the gene node features provided by NeDBIT. Let V be a set where each element $v_{i}$ is described by a feature vector $x_{i}$, with $x_{i}\in {\left[0,1\right]}^{d}$ representing a d-dimensional normalized feature vector computed by NeDBIT, and $y_{i}\in \{0,1\}$ denoting the initial binary label (1 for seed genes, i.e., the positive set P; 0 for unlabeled samples, i.e., the set U). The APU algorithm proceeds as follows:

Computed a similarity matrix, which is symmetric. Its elements $w_{ij}$ are defined as:

(15) $$\begin{array}{c} w_{ij}=\left\{\begin{array}{rr} 1-\frac{e_{ij}-m}{M-m}, & i\neq j\\ 1, & i=j \end{array}\right., \end{array}$$

where $e_{ij}=\sqrt{\sum_{k=1}^{d}(x_{i}^{k}-x_{j}^{k}{)}^{2}}$ represents the Euclidean distance between samples i and j in the k-th feature dimension computed by NeDBIT; $m={min}_{i,j}\{e_{ij}\}$ is the minimum distance among all pairs, and $M={max}_{i,j}\{e_{ij}\}$ is the maximum distance among all pairs.

The reduced similarity matrix $W_{r}$ is computed by filtering weak connections based on a threshold. Its elements are defined as:

(16) $$\begin{array}{c} w_{r,ij}=\left\{\begin{array}{cc} w_{ij}, & w_{ij}>q_{w}\\ 0, & otherwise \end{array}\right., \end{array}$$

where the threshold $q_{w}$ is set to a certain quantile of all elements in the matrix W (here, 0.8 [8]), used to exclude low-similarity connections during propagation. To construct a proper Markov process, $W_{r}$ is normalized as:

(17) $$\begin{array}{c} W_{n}=D^{-1}W_{r}, \end{array}$$

where D is a diagonal matrix whose diagonal elements $d_{ii}=\sum_{j}w_{r,ij}$ represent the sum of similarity connection strengths for node i.

Initialize the state vector of the propagation process, $g_{0}$. Let ∣P∣ denote the cardinality of the positive seed gene set P, and define the average feature vector $\hat{x}=({\hat{x}}^{1},\ldots ,{\hat{x}}^{d})$, where each dimension ${\hat{x}}^{k}=\frac{1}{|P|}\sum_{i\in P}x_{i}^{k}$ represents the average value of the seed genes in the k-th feature. The reliable negative genes are selected from those genes farthest from $\hat{x}$ in the feature space. To maintain class balance, the farthest ∣P∣ genes are chosen to form the reliable negative set RN. The i-th element of the state vector $g_{0}$ is defined as:

(18) $$\begin{array}{c} g_{0,i}=\left\{\begin{array}{cc} 1, & i\in \Sigma \\ -1, & i\in RN\\ 0, & otherwise \end{array}\right.. \end{array}$$

If a different number of reliable negative genes is selected, the initial value of *RN* genes in $g_{0}$ should be set to $-\frac{|P|}{|RN|}$ to ensure balanced initial values between positive and negative classes and that the sum of vector elements is zero.

Define a restart-based Markov propagation process. The propagation equation is given by:

(19) $$\begin{array}{c} g_{r}=\left(1-\alpha \right)W_{n}^{T}g_{r-1}+\alpha g_{0},r=1,2,\ldots , \end{array}$$

where the parameter α is set to 0.8 [8]. Starting from the initial state vector $g_{0}$, iteration continues until the difference between consecutive state vectors satisfies $∥g_{r}-g_{r-1}∥<10^{-6}$, at which point the process is considered to have reached the steady state $g_{\infty }$.

Only samples that do not belong to the positive set P or the reliable negative set RN are considered, and their values in $g_{\infty }$ are sorted. Based on the ranking, the remaining classes are assigned: in this study, the top 10% [8] are labeled as the likely positive (LP) class, and the rest are evenly distributed between weak negative (WN) and reliable negative (RN) classes.

In the final step, a classifier is trained for the ultimate prediction. The NeDBIT feature vector of each gene (comprising the four types of features: heat diffusion, balanced diffusion, NetShort, and NetRing) is used as the input, and the five pseudo-labels generated by APU are used as the target variables. A trained random forest (RF) model is then employed to predict, for each gene, the probability score of being a potential disease-associated gene.

**Figure 3:**
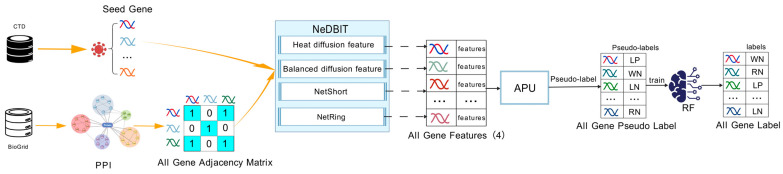
The workflow of NIAPU consists of the following steps: (i) acquisition and preprocessing of seed gene data and the protein–protein interaction (PPI) network; (ii) computation of NeDBIT features based on gene interaction edges extracted from the PPI network; (iii) application of the APU learning algorithm to score the entire gene set based on NeDBIT features, and preliminary classification of unlabeled genes into multiple categories (LP, WN, LN, RN) to form the RF training set, with seed genes always fixed as the P class; (iv) training of the model on the resulting multi-class training set, followed by classification and priority ranking; (v) performance evaluation of the prediction results using appropriate metrics [20].

In summary, the workflow of this module is illustrated in Figure 3.

### 2.3. Predicting SL Interacting Genes for NSCLC Potential Driver Genes

A growing number of GNN-based models have been proposed for the prediction of SL. The study by Feng et al. [42] demonstrated that, across different data scenarios and experimental settings, the SLMGAE model proposed by Hao et al. [26] achieved the best performance in multiple benchmark evaluations, confirming its effectiveness in capturing complex relationships among genes and integrating multi-source biological information. Therefore, in this study, we adopt the SLMGAE model as the core framework for our experiments, with the aim of integrating the latest available datasets while achieving high-accuracy prediction of SL gene pairs.

GO–Molecular Function (GO_MF) characterizes the functional properties of proteins or gene products at the molecular level and therefore provides important biological interpretability. In this study, we extend the original model by introducing a functional similarity view based on GO_MF as an additional supplementary perspective. In the original framework, the SL network adjacency matrix of genes was used as the primary view, while the semantic similarities derived from GO-Biological Process (GO_BP) and GO–Cellular Component (GO_CC), together with the PPI network, were incorporated as supplementary views. To further enhance the model’s capability to capture functional-level information, we employed the R package GOSemSim (version 2.30.0) [43] and adopted the semantic similarity measurement proposed by Wang et al. [44] to compute the functional similarity between any pair of genes in the GO_MF dimension. The resulting similarity matrix was integrated into the model as a newly added supplementary view.

The SLMGAE model employs a GCN as its encoder, and adopts the following propagation rule, as shown in Equation (20):

(20) $$\begin{array}{c} Z^{\left(l\right)}=LeakeyReLU\left(\hat{A}Z^{\left(l-1\right)}W^{\left(l\right)}\right), \end{array}$$

where

$$\hat{A}={\tilde{D}}^{-\frac{1}{2}}\tilde{A}{\tilde{D}}^{-\frac{1}{2}},\tilde{A}=I+A,LeakyReLU(x)=max(0.2x,x).$$

Here, $Z^{\left(l\right)}$ and $Z^{\left(l-1\right)}$ denote the output and input of the l-th GCN layer, respectively. The SL network adjacency matrix $A_{SL}$ is used as $Z^{\left(0\right)}$, serving as the initial input. $W^{\left(l\right)}$ represents the trainable weight matrix of the l-th layer; $\tilde{D}$ is a diagonal matrix whose diagonal elements are given by ${\tilde{D}}_{ii}=\sum_{j}{\tilde{A}}_{ij}$; A denotes the adjacency matrix of the current view; and I is the identity matrix.

Based on the two-layer GCN propagation defined in Equations (21) and (22), the node embedding matrix of the primary view, denoted as $Z_{2}^{SL}$, is obtained as follows:

(21) $$\begin{array}{c} Z_{SL}^{1}=LeakeyReLU\left(\hat{A}A_{SL}W_{1}^{SL}\right), \end{array}$$

(22) $$\begin{array}{c} Z_{SL}^{2}=LeakeyReLU\left(\hat{A}Z_{SL}^{1}W_{2}^{SL}\right). \end{array}$$

Furthermore, the graph structure of the primary view is reconstructed according to Equation (23):

(23) $$\begin{array}{c} S^{SL}=Z_{SL}^{2}W_{d}^{SL}{Z_{SL}^{2}}^{T}, \end{array}$$

where $W_{d}^{SL}$ is the trainable matrix of the decoder corresponding to this view.

Similarly, for each supplementary view u (1≤u≤4), the embedding matrix $Z_{u}^{\left(2\right)}$ and the reconstructed graph $S^{u}$ can be obtained using the same procedures defined in Equations (21)–(23). To ensure consistency and interaction across different views, the normalized propagation matrix $\hat{A}$ for each view is uniformly set to $A_{SL}$, while $Z^{\left(0\right)}$ is initialized using the adjacency or similarity matrix corresponding to each individual view.

Given the reconstructed graph matrix S and its corresponding original adjacency matrix Y($A_{SL}$), SLMGAE defines the reconstruction loss using the mean squared error (MSE), as shown in Equation (24):

(24) $$\begin{array}{c} MSE\left(Y,S\right)=\frac{2}{n\left(n-1\right)}\sum_{i=1}^{n}\sum_{j=i+1}^{n}{\left(Y_{ij}-S_{ij}\right)}^{2}. \end{array}$$

where n denotes the number of genes. Based on this, the reconstruction loss of the primary view, $S^{SL}$, is denoted as $L_{M}$, and the overall reconstruction loss of each supplementary view, $S^{u}$, is denoted as $L_{S}$:

$$L_{M}=MSE(A_{SL},S^{SL}),\;L_{S}=MSE(A_{SL},S^{u})\left(1\leq u\leq 4\right).$$

Note that the reconstructed matrices generated by Equation (23) are generally asymmetric, whereas $A_{SL}$ is symmetric. To avoid predicting two different values for the same SL interaction, SLMGAE computes the above losses by considering only the upper triangular part of the reconstructed matrices.

After reconstructing the score matrices of the primary and supplementary views, the model introduces an attention fusion layer to integrate the reconstructed score matrices from all views. For each supplementary view u (1≤u≤4), a weight ${\tilde{a}}_{ij}^{u}\in R^{n\times n}$ is randomly initialized for each node pair $\left(v_{i},v_{j}\right)$. Then, a softmax function is applied across different views for the same node pair to obtain the normalized attention matrix $a^{u}$ for view u. The element $a_{ij}^{u}$ is computed as follows:

(25) $$\begin{array}{c} a_{ij}^{u}=\frac{e^{{\tilde{a}}_{ij}^{u}}}{\sum_{x=1}^{4}e^{{\tilde{a}}_{ij}^{u}}}. \end{array}$$

The weighted similarity matrix of the supplementary views is subsequently calculated by:

(26) $$\begin{array}{c} W_{supp}=\sum_{u=1}^{4}a^{u}\circ S^{u}, \end{array}$$

where ∘ denotes element-wise multiplication. The primary view $S^{SL}$ and the weighted supplementary similarity $W_{\mathrm{supp}}$ are then fused to obtain the final predicted score matrix $S^{P}$:

(27) $$\begin{array}{c} S^{P}=S^{SL}+C\cdot W_{supp}, \end{array}$$

where C is a hyperparameter controlling the contribution of the supplementary views to the final prediction scores. The predicted score matrix $S^{P}$ is used for SL prediction, and the final prediction loss $L_{P}$ is measured by *MSE*:

(28) $$\begin{array}{c} L_{P}=MSE\left(A_{SL},S^{P}\right). \end{array}$$

Finally, the reconstruction losses—including the primary view loss $L_{M}$ and the supplementary view loss $L_{S}$—are combined with the prediction loss $L_{P}$ to construct the overall loss function:

(29) $$\begin{array}{c} L_{\mathrm{Total}}=L_{M}+\alpha L_{S}+\beta L_{P}, \end{array}$$

where α and β are hyperparameters controlling the relative contributions of $L_{S}$ and $L_{P}$ in the total loss.

**Figure 4:**
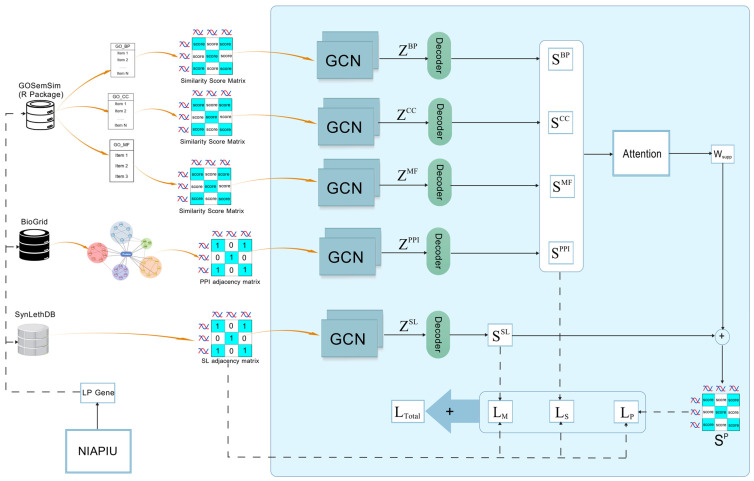
The architecture of the SLMGAE model and its application in the present study [20].

Adapted from the SLMGAE model architecture [26], the workflow of this module is illustrated in Figure 4.

### 2.4. Drug Repositioning Targeting Synthetic Lethal Genes of Potential NSCLC Driver Genes

Based on the concept of SL in cancer therapy, when a driver gene is aberrant or difficult to target directly, its SL-interacting genes can serve as alternative intervention points to achieve selective killing of cancer cells [45]. This module aims to predict drug targets for the SL-interacting genes of specific driver genes and to explore potential drugs that can act on these genes, thereby providing novel strategies and candidate options for overcoming drug resistance in NSCLC.

The SLMGAE model takes the association matrix as the primary view in a homogeneous network and introduces supplementary views as complementary sources of information, enabling the model to better capture relational structures and predict unknown associations in the graph, achieving promising performance. Therefore, in this study, we extend the application scope of this model from homogeneous networks to a heterogeneous graph setting. Specifically, it is employed to predict associations between genes and drugs in our task. In this context, we refer to the extended model as MVGAE.

In this study, the Canonical SMILES representations of drugs were obtained from the PubChem database via the PUG REST interface [46]. Subsequently, the SMILES strings were parsed and molecular structure objects were constructed based on the R package rcdk (version 3.8.2) [47], and the PubChem 881-bit substructure key fingerprints of drugs were generated using the R package fingerprint (version 3.5.7) [48]. The Tanimoto coefficient was then used to calculate the structural similarity between pairs of drug fingerprints, thereby constructing a drug structural similarity matrix [49]. The supplementary views of genes adopt the four supporting views used in the second module, namely PPI, GO_BP, GO_CC, and GO_MF.

To address the prediction task on a heterogeneous graph, the model makes slight adjustments to the input structure based on the propagation rules introduced in Section 2.3 For the main view of the network, is modified as follows:

$$A_{GD}=(\begin{array}{cc} 0 & A_{gd}\\ A_{gd}^{T} & 0 \end{array}),$$

where $A_{gd}\subset R^{g\times d}$ denotes the association matrix between genes and drugs, g is the number of genes, and d is the number of drugs. After adjustment, $A_{GD}\subset R^{\left(d+g)\times (d+g\right)}$. For each gene supporting view $A_{u}^{\prime }(1\leq u\leq 4)$, the adjustment is as follows:

$$A_{u}^{\prime }=(\begin{array}{cc} A_{u} & 0_{g\times d}\\ 0_{d\times g} & 0_{d\times d} \end{array}),$$

where $A_{u}\subset R^{g\times g}$ is the initial matrix of each supplementary view for genes. The supplementary view for drugs $A_{d}^{\prime }$ is defined as:

$$A_{d}^{\prime }=(\begin{array}{cc} 0_{g\times g} & 0_{g\times d}\\ 0_{d\times g} & A_{d} \end{array}),$$

where $A_{d}\subset R^{d\times d}$ is the drug similarity matrix obtained above.

By propagating the above networks in the MVGAE model according to Equations (21)–(29), the model is trained to obtain the optimal parameters.

### 2.5. Analysis and Screening in Network

**Figure 5:**
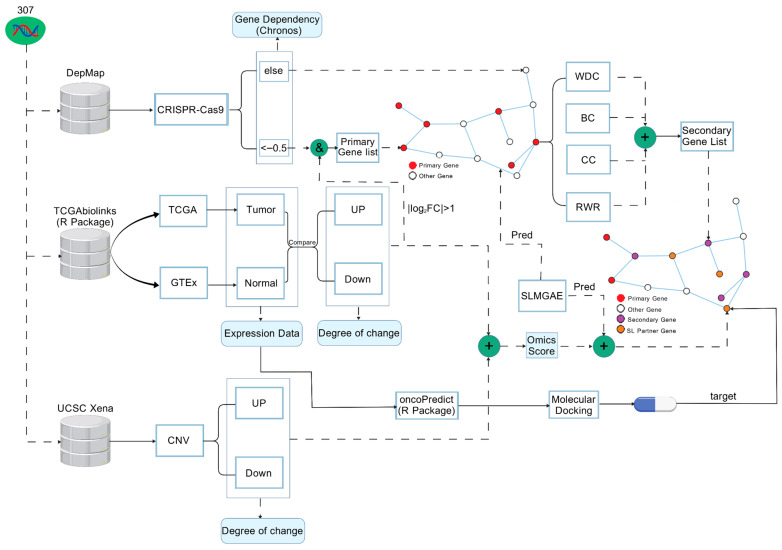
Workflow of this module [20].

In this module, the study will discuss the methods for screening and analyzing the results. The specific workflow of this module is shown in Figure 5.

Based on the R package TCGAbiolinks (version 2.32.0) [50,51,52], RNA-Seq transcriptome data (in Transcripts Per Million, TPM) and gene annotation for lung adenocarcinoma (LUAD) and lung squamous cell carcinoma (LUSC) were obtained from The Cancer Genome Atlas (TCGA) database (accessed on 13 October 2025). Both LUAD and LUSC are major subtypes of NSCLC, and in this study, they were integrated to form a unified NSCLC dataset. Meanwhile, RNA-Seq transcriptome data (in raw counts) and gene annotation of healthy lung tissue samples were obtained from the GTEx database using the R package recount3 (version 1.14.0) [53,54] (accessed on 13 October 2025). To unify expression units across different datasets and eliminate the effects of sequencing depth and library size, the normalization factor $s_{j}$ for each sample in GTEx was calculated following the method of DESeq2, defined as:

(30) $$\begin{array}{c} s_{j}={median}_{i}\left(\frac{k_{ij}}{g_{i}}\right), \end{array}$$

where $k_{ij}$ denotes the raw count of gene i in sample j, and $g_{i}$ is the geometric mean expression of gene i across its m samples, defined as:

(31) $$\begin{array}{c} g_{i}={\left(\sum_{j=1}^{m}k_{ij}\right)}^{1/m}. \end{array}$$

After normalization, the standardized expression value is calculated as:

(32) $$\begin{array}{c} n_{ij}=\frac{k_{ij}}{s_{i}}. \end{array}$$

To further correct for gene length and sequencing depth differences, the TPM expression value was calculated as:

(33) $$\begin{array}{c} {TPM}_{ij}=\frac{\frac{k_{ij}}{L_{i}}}{\sum_{i}\frac{k_{ij}}{L_{i}}}\times 10^{6}, \end{array}$$

where $L_{i}$ is the length of gene i in kilobases (kb). Using the above procedure, the raw RNA-Seq counts in GTEx can be converted to the same TPM expression format as TCGA. Finally, to approximate a normal distribution and be suitable for subsequent statistical tests, the TPM values from both datasets were log-transformed:

(34) $$\begin{array}{c} E_{ij}={log}_{2}\left({TPM}_{ij}+1\right). \end{array}$$

Divide the TCGA and GTEx samples into tumor and normal groups, respectively. Let the mean expression levels of each gene in the two groups be $E_{T}$ and $E_{N}$. The fold change (FC) of each gene is then defined as:

(35) $$\begin{array}{c} \log_{2}FC={AVE}_{T}/{AVE}_{N} \end{array}$$

A threshold $|{log}_{2}FC|$ of 1 was set [55], indicating a twofold difference in expression between the two groups, and genes exceeding this threshold were considered to have significant expression changes. In addition, a *t*-test was applied, with p<0.05 as the criterion for statistical significance, to assess whether the expression differences between the two groups were statistically significant.

The DepMap Portal is a large-scale database platform integrating human cancer gene dependency data [56] (version: 25Q3, accessed on 13 October 2025), including cell line dependency scores generated from *CRISPR-Cas9* gene knockout experiments (Chronos Score, CS) and other information. To exclude potential interference where gene expression differences might be caused by upstream factors rather than the gene’s own mutations, this study selected 165 NSCLC-related cell lines and evaluated the CS of genes in these cell lines. The score reflects the extent to which cell line survival depends on a given gene, and a threshold of −0.5 is commonly used to indicate that inhibition of the gene suppresses cell growth. Accordingly, genes with the mean CS across all cell lines (MCS) less than −0.5 [57] were selected as the primary gene list.

According to the biological principles of SL, the lethality caused by mutations in certain genes may be buffered through compensatory changes in other genes to maintain cell survival and adaptability. Therefore, the expression trends of SL gene pairs exhibit a certain degree of complementarity [58]. In addition, SL genes can show mutual exclusivity in certain features, such as DNA copy number variations (CNVs) [59]. It is assumed that the survival capability of these cells depends on or arises from the mutual exclusivity between two genes: if a cell simultaneously carries CNVs alterations in both genes, it will not survive. Thus, almost no surviving cells carry such simultaneous genetic events for the two genes.

In this study, CNVs data for genes in LUAD and LUSC were obtained from XENA [60] (accessed on 13 November 2025). These values were inferred from GISTIC2-normalized data [61] and reflect deviations of CN from normal values, standardized to the range [−2, 2], where negative values indicate deletions and positive values indicate amplifications. Only high-level amplifications and deletions, corresponding to +2 and −2, were considered as severe CN mutations [62], i.e., events that would not occur simultaneously in surviving cell lines.

A network was constructed with genes as nodes and the SL relationships predicted by the SLMGAE model as edges. The SL scores predicted by the SLMGAE model, the expression complementarity between the two nodes, and the CN mutual exclusivity were comprehensively considered as edge attributes. The calculation is as follows:

Let $E_{A}=\log_{2}{FC}_{A}$ and $E_{B}=\log_{2}{FC}_{B}$ denote the expression differences in gene A and gene B between normal and tumor tissues, respectively. The expression complementarity score between genes, ${EScore}_{AB}$, is defined as:

(36) $$\begin{array}{c} {EScore}_{AB}=\left\{\begin{array}{c} \begin{array}{cc} \left|E_{A}-E_{B}\right| & E_{A}\times E_{B}<0\\ 0 & else \end{array} \end{array}\right.. \end{array}$$

Let ${CScore}_{AB}$ denote the CN mutual exclusivity score between genes A and B, which is defined as:

(37) $$\begin{array}{c} {CScore}_{AB}=max\left(1-\frac{n_{AB}}{\min\left(n_{A},n_{B}\right)},0\right), \end{array}$$

where $n_{A}$ and $n_{B}$ are the numbers of samples in which gene A and gene B, respectively, exhibit CNVs events (high-level amplification or homozygous deletion), and $n_{AB}$ is the number of samples in which both gene A and gene B simultaneously have CNVs events.

After obtaining all EScore and CScore values for the edges, the weights of the two scores were calculated based on the principle of feature variance maximization: if a feature contributes more to distinguishing different gene pairs in SL relationships, it is assigned a higher weight. Specifically:

(38) $$\begin{array}{c} \beta =\frac{\sigma_{E}^{2}}{\sigma_{E}^{2}+\sigma_{C}^{2}},\;\;\gamma =\frac{\sigma_{C}^{2}}{\sigma_{E}^{2}+\sigma_{C}^{2}}, \end{array}$$

where $\sigma_{E}^{2}$ and $\sigma_{C}^{2}$ are the variances of EScore and CScore, respectively. β and γ denote the weights for EScore and CScore, respectively.

The multi-omics score of the SL relationship between gene A and gene B is then calculated as:

(39) $$\begin{array}{c} {Omics}_{AB}=\beta \times {EScore}_{AB}+\gamma {\times CScore}_{AB}. \end{array}$$

Next, we determined the fusion weight α by evaluating the statistical independence between the SL scores predicted by SLMGAE and the multi-omics scores. That is, the smaller the mutual information between the two variables, the more independent the information they provide, and the more balanced their combination should be. The calculation is as follows:

(40) $$\begin{array}{c} S_{rank}=rank\left(Score\right),O_{rank}=rank\left(Omics\right), \end{array}$$

where Score is the vector of SL scores predicted by SLMGAE, and Omics is the vector of multi-omics scores obtained above.

Then, B bootstrap resamplings were performed (In this study, we set B=200). In each iteration, n samples were drawn with replacement from the original data, and the mutual information was calculated:

(41) $$\begin{array}{c} I_{b}=I\left(S_{rank}^{b};O_{rank}^{b}\right),b=1,2,\ldots \ldots B, \end{array}$$

where I(X;Y) denotes the mutual information between variables X and Y. The average mutual information was computed as:

(42) $$\begin{array}{c} \bar{I}=\frac{1}{B}\sum_{i=1}^{B}I_{b}. \end{array}$$

Finally, the average mutual information was transformed into the fusion weight:

(43) $$\begin{array}{c} \alpha =\frac{1}{1+\bar{I}}, \end{array}$$

where α corresponds to the weight for the SLMGAE-predicted SL scores, and 1−α corresponds to the weight for the multi-omics scores. The final edge score between nodes in the network can thus be calculated as:

(44) $$\begin{array}{c} EdgeScore=\alpha \times Score+\left(1-\alpha \right)\times Omics=\alpha \times Score+\left(1-\alpha \right)\times \left(\beta \times EScore+\gamma \times CScore\right). \end{array}$$

In addition, we calculated the topological feature scores of genes in the primary list within the network based on mutual information and feature independence. Four topological features of nodes were considered: weighted degree centrality (WDC), betweenness centrality (BC), closeness centrality (CLC), and random walk with restart (RWR) scores, where the starting points of RWR were set as genes in the primary list and the restart probability was set to 0.3.

Construct the set:

(45) $$\begin{array}{c} F=\{WDC,BC,CLC,RWR\}. \end{array}$$

Compute:

(46) $$\begin{array}{c} X_{rank}=rank\left(X\right),X\in F. \end{array}$$

For each target feature T∈F, the sum of mutual information with all other features was computed:

(47) $$\begin{array}{c} {MI}_{T}=\sum_{F\in F\setminus \{T\}}I\left(T_{rank};F_{rank}\right). \end{array}$$

The independence score of each feature was then calculated as:

(48) $$\begin{array}{c} {\mathrm{Independence}}_{T}=\max\left({\mathrm{MI}}_{F}\right)-{\mathrm{MI}}_{T},F\in F \end{array}$$

The independence scores were normalized to obtain feature weights:

(49) $$\begin{array}{c} w_{T}=\frac{{\mathrm{Independence}}_{T}}{\sum_{F\in F}{\mathrm{Independence}}_{F}}. \end{array}$$

Finally, the integrated topological score for each gene was calculated as:

(50) $$\begin{array}{c} Topology\;Score=w_{D}\times WDC+w_{B}\times BC+w_{C}\times CLC+w_{R}\times RWR. \end{array}$$

Based on the integrated topological scores of genes in the primary gene list, this study selected the top 10% [63] ranked genes. These genes exhibit the highest structural centrality or propagation capability within the constructed SL network, have more potential informational connections with other genes, and may play more critical roles in the pathogenesis of NSCLC. The parameter values are provided in Supplementary Matarials, Table S1. Therefore, these genes were designated as the secondary gene list. Based on the edge scores in the network, the two highest-scoring connected nodes for each gene were selected as their SL interacting partners.

Based on the gene–drug associations predicted by the MVGAE model, the top 10% of drugs (ranked by predicted score [63]) for each gene in secondary gene list were selected for further screening and analysis. oncoPredict is an R package (version 1.2) for drug response prediction and drug–gene association analysis [64]. In this study, we used oncoPredict to evaluate the responses of genes to drugs. Its built-in CTRP2 dataset, containing 51,847 genes, 829 cell lines, and 545 drugs, was used as the training set.

Using the trained model, we calculated the correlation coefficients between gene expression in NSCLC (RNA-Seq data in TPM) and drug sensitivity. A larger absolute value of the correlation coefficient indicates a stronger association between gene expression and drug sensitivity, while the sign indicates the direction of the association: positive values indicate that higher gene expression enhances drug sensitivity, and negative values indicate the opposite.

For preliminary drug screening based on gene expression trends in normal and tumor tissues: if a gene is upregulated in tumor tissues, drugs whose sensitivity is positively correlated with its expression are prioritized; if a gene is downregulated, drugs whose sensitivity is negatively correlated with its expression are preferred.

## 3. Results

### 3.1. Results of NIAPU

The 144 seed genes screened and obtained from the CTD database were used as the P set. Meanwhile, the 18,678 genes and 878,761 PPI interaction edges collected in BioGrid 4.4 were used as the raw input for feature engineering. Based on this, the NeDBIT feature representations of genes, along with their corresponding APU scores and initial pseudo-labels, were obtained to train the RF model. The dataset was split into an 80% training set (13,074 genes) and a 20% test set (5604 genes) (The training and testing methods followed those described in Ref. [9]). for multi-class classification training and validation. The RF model ultimately assigned predicted labels to each gene, enabling the selection of a candidate list of potential NSCLC driver genes.

**Figure 6:**
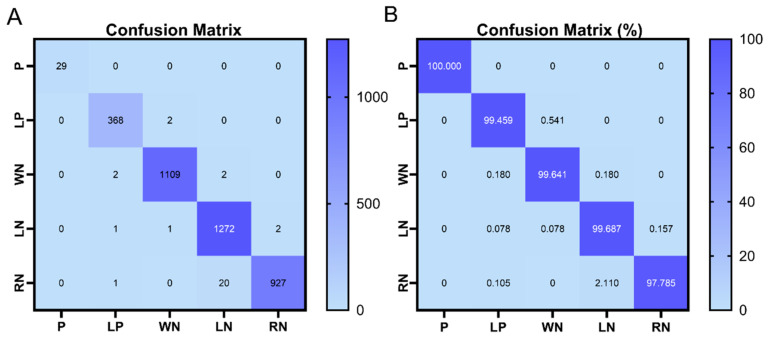
(**A**) Confusion matrix of the model’s classification performance; (**B**) Proportion of correctly classified samples by the model.

On the test set, the performance of the RF model for the multi-class task is shown in the confusion matrix in Figure 6. After training the RF model using pseudo-labels generated by the NIAPU model, the model effectively distinguished P-class genes from other categories. Moreover, for the pseudo-classes LP, WN, LN, and RN, the model also achieved excellent classification performance.

Five-fold stratified cross-validation was conducted to evaluate the model, ensuring that the class distribution in each fold’s training and test sets was consistent. Precision, Recall, and F1-score for each class were used as evaluation metrics, and the results are summarized in Table 2.

The trained model was applied to the whole-genome data to assign corresponding labels to all genes (with the seed genes fixed as P-class). Subsequently, the predicted full-labeled gene set was further analyzed: LP-class genes were ranked according to their predicted association scores, and 1850 genes were ultimately identified as potential NSCLC driver genes, among which 1796 genes overlapped with the 16,786 genes extracted and filtered from the CTD database based on literature reports or other computational prediction methods.

TFLINK is a database integrating a large number of experimentally validated or high-confidence predicted transcription factor (TF)–target gene regulatory relationships, which can be used to identify whether candidate genes are co-regulated by TFs closely associated with tumor initiation and progression. To further validate the reliability of the model predictions, transcription factor regulatory analysis was performed using the TFLINK database integrated through the DAVID platform [65,66]. The analysis results are shown in Table 3.

### 3.2. Results of SLMGAE

In Section 3.1, we identified a set of potential key genes associated with NSCLC that may play important roles in cancer initiation and progression. However, directly inhibiting or targeting these genes often faces challenges such as undruggability and drug resistance [73,74]. To overcome the poor efficacy caused by these issues, this study introduces the concept of SL, aiming to achieve selective killing of cancer cells by targeting the SL partner genes of the target genes. This strategy can bypass resistance or undruggability of the original targets, providing new avenues for precision therapy in NSCLC [75].

For example, the tumor suppressor gene SMARCA4 is frequently inactivated in NSCLC and is difficult to target directly due to its structural characteristics. Studies have shown that SMARCA4 loss leads to a significant reduction in cyclin D1 levels, rendering tumor cells highly sensitive to CDK4/6 inhibitors. Based on this, SMARCA4 and CDK4/6 form an SL gene pair. Inhibiting CDK4/6 can effectively kill SMARCA4-deficient tumor cells, offering new potential targets and drug intervention strategies for NSCLC treatment [76].

P-class genes, as clinically validated seed genes, have been extensively explored in terms of their biological information. In contrast, LP-class genes—particularly the 1850 potential driver genes screened above—may play roles in NSCLC that are not yet fully understood. Identifying their SL partner genes could reveal novel lethal pathways and provide more feasible therapeutic strategies for clinical application. Therefore, this study focuses on the 1850 LP-class genes as the main subjects of investigation.

We used five-fold stratified cross-validation to evaluate the generalization performance of the model (The training and testing methods followed those described in Ref. [26]). The model’s performance was assessed using AUC, AUPR, Recall, Precision, and F1-Score, as shown in Table 4.

The experimental results indicate that, on a 1:1 balanced test set of positive and negative samples (positive samples refer to gene pairs with known SL interactions, while negative samples refer to gene pairs without known SL interactions.), the model demonstrates strong generalization ability, with performance metrics across folds being very close. Therefore, the model is well-suited for the tasks in this study.

To further evaluate whether incorporating GO_MF information as an additional supporting view can enhance the model’s ability to capture potential relationships between genes, we conducted five-fold cross-validation experiments on the same dataset with identical data size, positive-to-negative sample ratio, and hyperparameter settings. We compared the original SLMGAE model without GO_MF information to the improved model that includes GO_MF as an additional supporting view.

**Figure 7:**
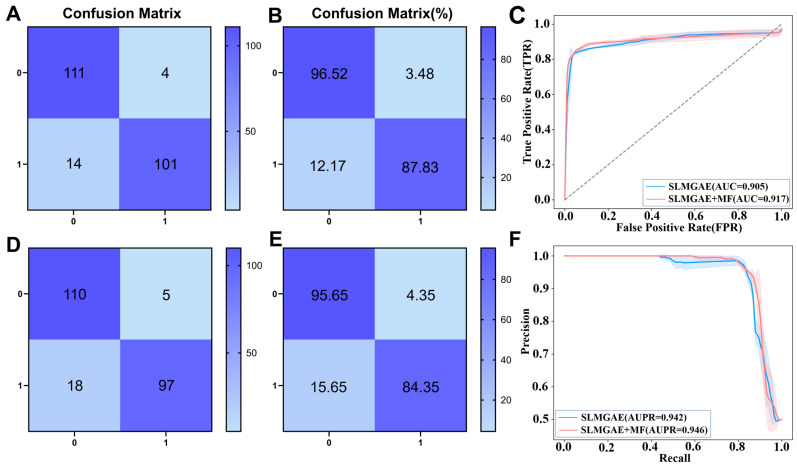
Performance comparison between the two models with and without the GO_MF supporting view. (**A**,**B**) show the confusion matrix and the proportion of correctly classified samples for the improved model with the GO_MF view, while (**D**,**E**) present the corresponding results for the original model. (**C**,**F**) display the PR curves and ROC curves of the two models under five-fold cross-validation (mean ± standard deviation).

For each fold, the resulting Receiver Operating Characteristic (ROC) and Precision-Recall (PR) curves were standardized and averaged: the vertical coordinates of each fold’s curve were mapped to a unified grid using linear interpolation, and the mean and standard deviation were calculated for each horizontal coordinate point. As shown in Figure 7, the model with the GO_MF supporting view achieved improvements across all metrics, particularly in predicting positive samples, compared to the original model. Additionally, the standard deviations of the PR and ROC curves decreased, producing smoother and more stable curves, with higher AUC and AUPR values. The average and maximum standard deviations of the PR and ROC curves for the models with and without GO_MF integration are provided in Supplementary Materials, Table S15. These results demonstrate that the molecular functional information contained in the GO_MF data introduced in this study is effective and valuable for enhancing model predictions.

Finally, based on the improved model, we applied it to the prediction task on the set of 1850 genes to identify potential SL interaction pairs. The model predicted a total of 1,709,753 SL gene pairs. These pairs were ranked according to the prediction scores, and those with scores above the 99.9th percentile [26]) (0.09195) were selected. As a result, 1710 potential SL interaction pairs involving 307 genes were retained for subsequent analyses.

### 3.3. Results of MVGAE

In this section, we constructed a heterogeneous gene–drug interaction graph using the 307 genes identified in Section 3.2 and the 1263 drugs collected from DGIDB v5.0.10 (Section 2.1), along with 1734 interaction edges between them. This graph was then used as the input dataset for training the MVGAE model.

To assess the generalization capability of the model, we performed five-fold stratified cross-validation (The training and testing methods are the same as in Section 3.2.). Model performance was evaluated using AUC, AUPR, Recall, Precision, and F1-Score, and the results are reported in Table 5.

We compare the performance of the DGAMGAE model on our dataset with several representative baseline methods. The models included in the comparison are:

- ADADR [77]: An adaptive GCNS-based method that deeply integrates node features with topological structure.
- GC-MC [78]: A graph auto-encoder framework that performs message passing on a bipartite graph representing interaction data.
- LRGCPND [79]: This model adopts a sequential strategy to model the bipartite graph of ncRNA drug resistance. It first aggregates neighborhood information to obtain node representations, then performs feature transformation through linear operations, and finally makes predictions using residual connections.
- DGCL [80]: A novel dynamic hypergraph contrastive learning framework that applies graph convolution to capture explicit local relationships between drugs and genes, while the combination of dynamic hypergraph structure learning and hypergraph message passing enables the aggregation of information at a global level.
- NIMCGCN [81]: A novel neural inductive matrix completion method based on graph convolutional networks for predicting associations between miRNAs and diseases.

**Figure 8:**
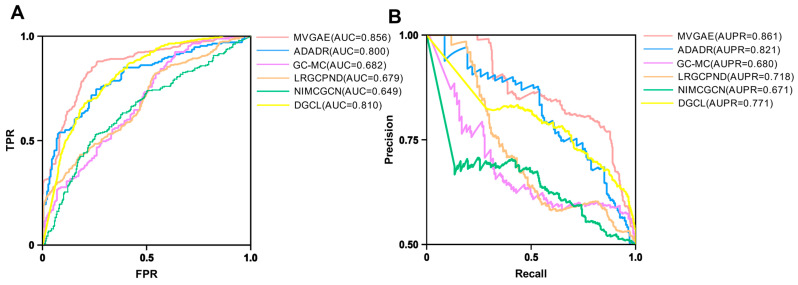
(**A**) ROC Curve of Comparative Experimental in 5-fold CV. (**B**) PR Curve of Comparative Experimental in 5-fold CV.

All models were trained and evaluated on the same dataset using five-fold cross-validation. The ROC and PR curves obtained from the comparative experiments are shown in Figure 8.

In summary, the multi-view MVGAE model demonstrates strong performance even on sparse bipartite graphs with limited information. Compared with other models, MVGAE achieves significantly higher AUC and AUPR values. Applying this model to our dataset, we selected 305,832 gene–drug association edges with predicted scores greater than 0 as potential targetable drugs for the genes.

### 3.4. Analysis of the Three Models and Network

Following Equations (30)–(35), we obtained 70 primary list genes from the 307 genes. Based on Equations (36)–(50), we further identified 7 secondary list genes along with their

**Figure 9:**
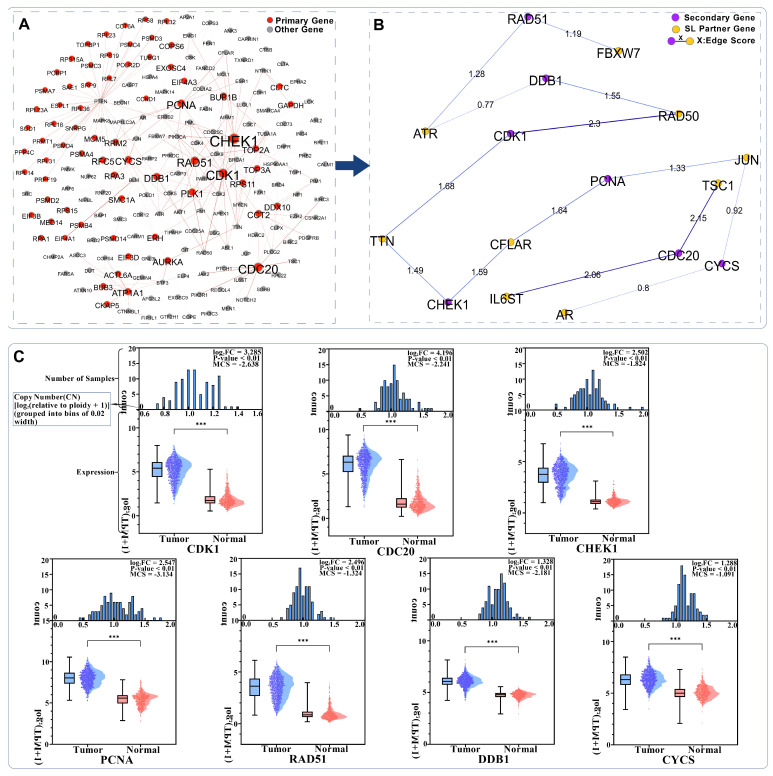
(**A**): Network topology distribution between the primary gene list and other genes; (**B**): Network topology of the secondary gene list and their SL interacting genes selected as described above, where the values on the edges represent the integrated edge scores; (**C**): Multi-omics data of the seven secondary genes, including DNA copy number (CN) (raw values transformed by log_2_(relative to ploidy + 1); to facilitate analysis of the distribution of gene CN in NSCLC cell lines, the values were binned at equal intervals of 0.05) and mean Chronos Score (MCS), which represents the average Chronos Score of each gene across all NSCLC samples. *** represents *p*-value < 0.01.

2 SL interaction partners each. The network topology of the genes in the two lists, as well as the multi-omics data for the secondary gene, are shown in Figure 9.

The seven secondary genes and their respective SL interacting genes are as follows: CDK1 (RAD50, TTN), CDC20 (TSC1, IL6ST), PCNA (CFLAR, JUN), CHEK1 (TTN, CFLAR), DDB1 (RAD50, ATR), RAD51 (ATR, FBXW7), and CYCS (JUN, AR). The nine genes that have SL interactions with these secondary genes are selected as the focus for subsequent analysis.

Based on the gene-related drugs screened using the method described in Section 2.5, this study obtained the experimentally resolved PDB protein crystal structures corresponding to the target genes from UniProt [82,83] to serve as receptors. Meanwhile, the three-dimensional structures of the corresponding drugs were retrieved from DrugBank [84,85] to serve as small-molecule ligands for subsequent analyses. The relevant information is summarized in Table 6.

**Figure 10:**
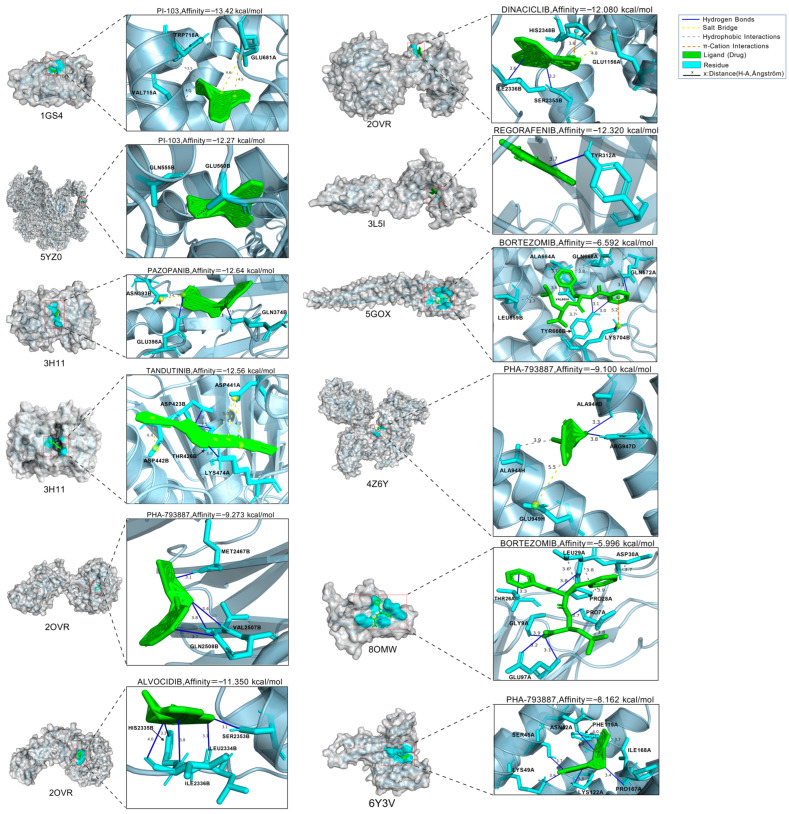
Docking results between the PDB crystal structures of target genes and their corresponding drugs.

This study employed AutoDock Vina v1.2.7 [86,87] to perform molecular docking simulations between the aforementioned receptors and their corresponding crystal structures. The conformation with the lowest binding affinity was selected as the docking result. Docking outcomes were further processed and visualized using *PyMOL* (version 3.1.6.1) [88] and *PLIP* (version 3.0.0) [89,90]. Detailed results are presented in Figure 10. The results of the PLIP-based interaction analysis corresponding to Figure 10 are shown in the Supplementary Materials, Tables S2–S13.

Based on the molecular docking results, the predicted candidate drugs were able to form multiple stable interaction modes with their corresponding receptors, including hydrogen bonds, salt bridges, and π–π interactions. The docking conformations exhibited favorable binding energies, suggesting potential stable interactions between the ligands and receptors. These computational binding modes provide a structural rationale for the predicted associations and serve as a basis for prioritizing compounds for further experimental testing.

It is important to note that the molecular docking simulations and expression-sensitivity correlation analyses conducted in this module serve as computational filters for prioritization. While these in silico evidences provide initial support for the potential binding affinity and biological relevance of the candidates, they are not substitutes for experimental validation. Therefore, the following screening steps are designed to narrow down the candidate list for subsequent experimental investigation.

We searched ClinicalTrials.gov [91] for clinical trials evaluating the above drugs in the treatment of NSCLC patients to preliminarily assess their actual efficacy. The relevant trials for each drug are summarized in Table 7.

Based on experimental research data, several of the drugs predicted in this study have already been tested in NSCLC treatment trials and have completed various clinical phases, indicating their potential value in treating NSCLC. Notably, PAZOPANIB demonstrated positive therapeutic effects and had its trial phase terminated early, further highlighting its promising clinical potential. Based on these findings, it is recommended to advance the drugs that have shown preliminary efficacy to the next clinical phase.

Additionally, a few drugs were discontinued due to limited efficacy or other reasons. Considering the theoretical evidence provided in this study, further in-depth research is suggested for these drugs to validate their potential value. Overall, most of the drugs discussed in this study have made progress in clinical trials and exhibit certain therapeutic potential. Therefore, for drugs that have not yet undergone trials (e.g., PI-103, PHA-793887, TANDUTINIB), the results of this study suggest that they also possess significant research value, particularly those repeatedly highlighted in our findings, such as PHA-793887, which merit further investigation.

## 4. Discussion

The identification of key driver genes is fundamental to precision oncology in NSCLC. While conventional targeted therapies focus on these critical drivers, their clinical efficacy is frequently hampered by the non-druggability of certain targets and the rapid emergence of adaptive resistance [74,75]. Furthermore, the de novo development of targeted inhibitors is often stymied by prohibitive costs and protracted development cycles, failing to keep pace with the evolving mutational landscape of NSCLC [27].

To circumvent these clinical bottlenecks, we developed an integrated computational framework that bridges SL with drug repurposing. Unlike prior studies that address driver gene identification, SL prediction, or drug screening in isolation, our work establishes a unified, end-to-end analytical pipeline specifically tailored for NSCLC. This systematic integration enables a seamless transition from biological pathogenesis to targeted therapeutic intervention. Methodologically, a key innovation of this study is the cross-domain adaptation of the SLMGAE model, transitioning it from SL interaction prediction to the drug repurposing task (redefined as MVGAE). This approach leverages the synergy of multi-omics data to capture complex gene-drug associations, offering a robust and scalable strategy for the rational design of precision oncology. Furthermore, we conducted a thorough analysis and filtering of the computational results based on multi-omics data. By constructing a gene interaction network combined with SL mechanisms, we predicted a set of the most promising NSCLC driver genes and their SL partner genes. Integrating drug sensitivity data, we predicted and screened corresponding targeted drugs. Finally, molecular docking analyses were performed to assess the potential binding stability and interaction patterns between candidate drugs and the PDB crystal structures of candidate therapeutic target gene-encoded proteins. The results provide supportive in silico evidence that the predicted drugs may form stable complexes with their corresponding targets, thereby offering additional rationale for drug repositioning.

As a computational prediction, our framework predicted *RAD51* and *ATR* as a candidate SL gene pair (see Section 3.2). This result should be strictly regarded as a hypothesis requiring future experimental validation. Encouragingly, existing literature provides conceptual support that is consistent with this prediction. Previous experimental studies have reported that loss of *RAD51* impairs homologous recombination and increases replication stress, thereby rendering cells highly dependent on ATR/CHK1-mediated checkpoint signaling. Under such conditions, *ATR* inhibition has been shown to induce SL-like effects [92]. The agreement between our computational prediction and prior biological findings supports the plausibility of this candidate interaction. Similarly, several compounds prioritized by our model (e.g., PAZOPANIB, BORTEZOMIB) have previously entered clinical evaluation for NSCLC (Table 7). This observation suggests that the ranking framework is capable of recovering biologically meaningful associations. Nevertheless, these findings should be interpreted as consistency with existing evidence rather than confirmation of newly predicted interactions. Furthermore, among the seven predicted potential NSCLC driver genes (secondary gene list), *CDK1*, *CDC20*, *CHEK1*, and *RAD51* have been reported to be significantly associated with NSCLC prognosis in previous studies [93,94,95]. This literature overlap further supports the biological relevance of the model’s outputs, though functional validation of the newly proposed candidates remains strictly necessary. Taken together, integrating existing experimental evidence with our computational predictions, it can be concluded that the computational framework constructed in this study—based on SL mechanisms and drug repositioning—offers substantial practical guidance for advancing NSCLC therapeutic research. Furthermore, genes and drugs predicted in this study that have not yet been widely reported or experimentally validated may also hold potential therapeutic value, warranting further biological and clinical investigation in subsequent studies.

According to the computational framework established in this study, the SL interactions identified from multi-omics data can, in a strict sense, be considered an important extended category of the SL mechanism: synthetic dosage lethality (SDL) [45]. In this mode of action, when a gene is overexpressed or functionally hyperactivated, the inactivation of its SDL partner gene becomes lethal. Targeting this SDL partner gene can therefore induce selective death of cancer cells.

The results of this study not only provide concrete and feasible strategies for NSCLC treatment, but also highlight the significant guiding value of a computational framework that integrates multi-source data, multiple models, and diverse methodologies in cancer research. Building on this foundation, future work will focus on further validating the results obtained in this study, while also innovating and refining the computational framework and extending its application to broader research scenarios, with the ultimate goal of offering systematic and scalable computational support for precision therapy across different cancer types.

Despite providing valuable insights and methodological directions for NSCLC treatment, this study has certain limitations:

First, the SL mechanism may involve complex interactions among multiple genes, whereas the present study only considers pairwise SL interactions and does not yet account for higher-order multi-gene interactions.

Second, the conclusions are primarily derived from theoretical analyses and integrative multi-omics computations; the molecular mechanisms in real biological systems may be more complex.

Third, selecting initial seed genes from the CTD database introduces potential curation bias. The 144 positive seed genes, derived from manually curated ‘Marker/Mechanism’ or ‘Therapeutic’ annotations, are susceptible to publication bias, historical research focus, and annotation lag. Consequently, the model’s feature space may skew toward well-characterized genes, potentially limiting its generalization to understudied but biologically relevant candidates. To mitigate this, our subsequent multi-omics screening—incorporating differential expression, gene dependency, and network topology—provides orthogonal, literature-independent evidence. While this approach effectively counterbalances initial biases, we acknowledge they cannot be completely eliminated; thus, our predictions should be interpreted as high-confidence candidates requiring experimental validation.

Fourth, dependence on knowledge bases and potential circularity. The framework’s performance inherently relies on the quality of the underlying databases (Section 2.1). While strict data splitting and masking during cross-validation effectively mitigate the risk of circularity from overlapping sources, future work must incorporate independent external cohorts and experimental validation.

Fifth, generalizability beyond NSCLC. Although the mathematical framework is conceptually extensible, the current data were specifically tailored for NSCLC. Direct transferability to other malignancies (e.g., breast or colorectal cancer) requires future empirical validation to assess broader applicability.

Although our framework utilizes patient-specific multi-omics profiles to address inter-individual variability, relying on bulk sequencing data remains a limitation. Bulk data captures average molecular signals, inevitably masking the genetic variations in specific subclonal populations that often drive adaptive drug resistance. To achieve true personalized precision, future studies should integrate single-cell RNA sequencing (scRNA-seq) data. Transitioning to single-cell analysis will enable our mathematical models to decipher intra-tumoral heterogeneity and refine SL predictions at the cellular level.

Nevertheless, the findings still carry strong guiding significance and can serve as a basis for prioritizing candidate genes and drugs for subsequent experimental studies, thereby narrowing the research scope and improving efficiency.

In future work, we plan to explore several approaches to further reduce curation bias and improve generalizability. (i) integrating multiple independent seed gene sources (e.g., GWAS catalog, COSMIC, ClinVar) to create a more diverse and representative positive set; (ii) applying transfer learning techniques to leverage knowledge from well-studied genes while enabling discovery of novel candidates; and (iii) integrating unsupervised or semi-supervised learning approaches that rely less on curated positive labels. Additionally, validating our framework on independent cancer types would help assess its generalizability beyond NSCLC.

Additionally, the framework could be extended to model NSCLC drug resistance by integrating dynamic transcriptomic changes, acquired mutation profiles, and pathway adaptations. Furthermore, incorporating protein structural features and protein–drug binding properties would enhance mechanistic interpretability and target prioritization. Together, these extensions provide a comprehensive representation of resistance evolution, thereby strengthening the model’s translational potential.

## 5. Conclusions

Based on the computational framework proposed in this study, we have predicted a set of candidate genes and drugs for further investigation in NSCLC. These include potential driver genes (*CDK1*, *CDC20*, *PCNA*, *CHEK1*, *DDB1*, *RAD51*, *CYCS*), their predicted SL partners (e.g., *RAD50*, *TTN*, *TSC1*, *IL6ST*, *CFLAR*, *JUN*, *ATR*, *FBXW7*), and corresponding repurposed drug candidates (e.g., BORTEZOMIB, PHA-793887, PI-103). The complete list of predicted interactions and supporting evidence is provided in Supplementary Table S14, which summarizes the source, score, and relevant literature for each finding. While these findings provide a strong rationale supported by multi-omics data and structural bioinformatics, they remain predictive and require rigorous experimental validation through in vitro and in vivo studies to confirm the therapeutic potential of these candidate gene-drug pairs. While the current study provides a computational foundation, subsequent experimental efforts—including CRISPR validation, in vitro testing in NSCLC models, pharmacological evaluation, and drug docking validation—will be essential to establish the therapeutic relevance of the identified SL pairs.

## Figures and Tables

**Table 1:** Summary of datasets and statistical characteristics used in this study.

	*BioGrid v4.4*	CTD	*SynLethDB v3.0*	*DGIDB v 5.0.10*
Number of Genes	18,678	144	-	-
PPI Interactions	878,761	-	-	-
SL Interactions	-	-	22,966	-
Number of Drugs	-	-	-	1263
Drug–Gene Associations	-	-	-	1734

**Table 2:** Performance of the RF model in 5-fold cross-validation (Average±Std).

Label	Precision	Recall	F1-Score
P	1.000 ± 0.000	1.000 ± 0.000	1.000 ± 0.000
LP	0.995 ± 0.005	0.987 ± 0.009	0.991 ± 0.007
WN	0.996 ± 0.003	0.997 ± 0.002	0.996 ± 0.002
LN	0.990 ± 0.003	0.993 ± 0.001	0.992 ± 0.002
RN	0.996 ± 0.001	0.995 ± 0.002	0.995 ± 0.001
Macro acv	0.995 ± 0.002	0.994 ± 0.002	0.995 ± 0.002
Weighted avg	0.994 ± 0.002	0.994 ± 0.002	0.994 ± 0.002
Accuracy	0.994 ± 0.002	-	-

**Table 3:** TFLINK transcription factor enrichment analysis results. Transcription Factor: Name of the significantly enriched transcription factor; Enrichment Ratio (RT): Proportion of the 1850 genes regulated by the transcription factor; *p*-value: Statistical significance of the enrichment result; FDR: False Discovery Rate; Benjamini: Correction for multiple hypothesis testing; Association: Overview of the transcription factor’s biological role in NSCLC development and progression; Evidence: Literature references supporting the transcription factor’s relevance to NSCLC.

Transcription Factor	RT	*p*-Value	FDR	Benjamini	Association	Evidence
TP53 (Tumor protein p53)	95.14%	$8.70\times 10^{-67}$	$1.17\times 10^{-66}$	$2.91\times 10^{-66}$	Associated with poor survival in NSCLC patients who undergo immunotherapy	Zhao et al., 2016 [67]
c-MYC (MYC proto-oncogene)	95.84%	$1.64\times 10^{-32}$	$2.03\times 10^{-32}$	$3.66\times 10^{-32}$	disrupts the normal balance of cell growth and proliferation	Sunpaweravon et al., 2022 [68]
STAT3 (Signal transducer and activator of transcription 3)	93.24%	$1.38\times 10^{-91}$	$2.18\times 10^{-91}$	$6.88\times 10^{-91}$	frequently observed in NSCLC, and high expression levels of STAT3 in resected tumor samples are associated with poor prognosis.	Harada et al., 2014 [69]
HIF1A (Hypoxia inducible factor 1 subunit alpha)	94.76%	$3.96\times 10^{-80}$	$5.48\times 10^{-80}$	$1.62\times 10^{-79}$	inhibiting the Hippo-YAP pathway, which in turn suppresses ferroptosis.	Zheng et al., 2023 [70]
RUNX2 (RUNX family transcription factor 2)	87.78%	$3.20\times 10^{-99}$	$1.59\times 10^{-99}$	$1.88\times 10^{-98}$	as a key regulatory factor and potential biomarker for the diagnosis and treatment of lung cancer.	Otálora et al., 2022 [71]
E2F1 (E2F transcription factor 1)	95.68%	$1.12\times 10^{-95}$	$1.93\times 10^{-95}$	$6.12\times 10^{-95}$	Promotes NSCLC progression by activating the PI3K/AKT pathway through MCM4	Cai et al., 2025 [72]

**Table 4:** Performance of the SLMGAE model for this task under five-fold cross-validation.

	AUC	AUPR	Recall	Precision	F1-Score
Fold1	0.9114	0.9407	0.8435	0.9604	0.8981
Fold2	0.9130	0.9359	0.8349	0.9697	0.8972
Fold3	0.9201	0.9532	0.8609	0.9802	0.9167
Fold4	0.9230	0.9542	0.8596	0.9800	0.9159
Fold5	0.9161	0.9434	0.8596	0.9159	0.8869
Average	0.9167	0.9459	0.8517	0.9612	0.9030

**Table 5:** Performance of the MVGAE model for this task under five-fold cross-validation.

	AUC	AUPR	Recall	Precision	F1-Score
Fold1	0.8260	0.8319	0.8473	0.7313	0.7850
Fold2	0.8467	0.8513	0.8559	0.7577	0.8038
Fold3	0.8313	0.8475	0.8156	0.7201	0.7649
Fold4	0.8481	0.8476	0.8790	0.7457	0.8069
Fold5	0.8563	0.8609	0.8786	0.7696	0.8205
Average	0.8417	0.8478	0.8553	0.7449	0.7962

**Table 6:** Predicted Gene–Drug Associations with Expression–Sensitivity Correlation (Gene: Target gene (SL partner genes of secondary list genes); PDB ID: Experimentally resolved PDB protein crystal structure ID of the target gene; Drug/DrugBank ID: Predicted drug and its corresponding ID in DrugBank; Cors: Spearman correlation between the gene expression level and drug sensitivity; *p*-value: Statistical significance of the correlation).

Gene	PDB ID	Drug	Drugbank ID	Cors	*p*-Value
*AR*	1GS4	PI-103	DB17046	−0.310	$7.270\times 10^{-25}$
*ATR*	5YZ0	PI-103	DB17046	−0.556	$1.454\times 10^{-86}$
*CFLAR*	3H11	PAZOPANIB TANDUTINIB	DB06589 DB05465	−0.285 −0.335	$3.627\times 10^{-21}$ $4.890\times 10^{-29}$
*FBXW7*	2OVR	PHA-793887 ALVOCIDIB DINACICLIB	DB12686 DB03496 DB12021	−0.547 −0.601 −0.466	$3.510\times 10^{-83}$ $9.001\times 10^{-105}$ $5.798\times 10^{-58}$
*IL6ST*	3L5I	REGORAFENIB	DB08896	−0.561	$1.988\times 10^{-88}$
*RAD50*	5GOX	BORTEZOMIB	DB00188	−0.480	$1.042\times 10^{-61}$
*TSC1*	4Z6Y	PHA-793887	DB12686	−0.349	$1.317\times 10^{-31}$
*TTN*	8OMW	BORTEZOMIB	DB00188	−0.504	$6.200\times 10^{-69}$
*JUN*	6Y3V	PHA-793887	DB12686	−0.516	$9.841\times 10^{-73}$

**Table 7:** Specific clinical trials and their statuses for the relevant drugs in the treatment of NSCLC.

Drug.	Status	Trial Count	Clical Sparse	Terminated Reason
PAZOPANIB	Completed Terminated	8 6	I, II, III I, II	- Get positive results (1); Other resons ^1^ (5).
ALVOCIDIB	Terminated	1	I	Not disclosed.
DINACICLIB	Completed	2	II	-
REGORAFENIB	Completed Terminated	2 1	II I	- Not disclosed.
BORTEZOMIB	Completed Terminated	14 7	I, II I, II	- Insufficient efficacy (2); Other reasons (5).

^1^ Other reasons: Including non-therapeutic factors such as: low participant recruitment, slow trial progress, and decisions made by review committees.

## Data Availability

The data presented in this study are available in publicly available repositories. Specifically, data were sourced from the following: 1. Gene data related to non-small cell lung cancer (NSCLC) were accessed from the Comparative Toxicogenomics Database (CTD) (https://ctdbase.org/) (accessed on 19 September 2025) [33,34]. 2. Human protein–protein interaction (PPI) data were retrieved from BioGRID version 4.4 (https://thebiogrid.org/) (accessed on 11 September 2025) [35,36]. 3. Synthetic lethal (SL) interaction data were obtained from SynLethDB 3.0 (https://synlethdb.sist.shanghaitech.edu.cn/) (accessed on 11 September 2025) [37]. 4. Gene-drug interaction data were collected from the Drug-Gene Interaction Database (DGIDB v5.0.10) (doi:10.1093/nar/gkad1040) (accessed on 10 September 2025) [38,39]. 5. RNA-seq transcriptome data, gene annotation, and clinical information for lung adenocarcinoma (LUAD) and lung squamous cell carcinoma (LUSC) were obtained from The Cancer Genome Atlas (TCGA) database via the R package TCGAbiolinks (references: doi:10.1093/nar/gkv1507; doi:10.12688/f1000research.8923.2; doi:10.1371/journal.pcbi.1006701) (reference number) (accessed on 13 October 2025) [50,51,52]. 6. RNA-seq transcriptome data for healthy lung tissues were obtained from the GTEx database via the R package recount3 (http://bioconductor.org/packages/recount3/) (accessed on 13 October 2025) [53,54]. 7. Cell line dependency scores from CRISPR-Cas9 experiments were accessed from the DepMap Portal (https://depmap.org/portal/) (version: 25Q3, accessed on 13 October 2025) [56]. 8. Copy number variation (CNV) data for genes in LUAD and LUSC were retrieved from the UCSC Xena platform (doi:10.1186/s13062-015-0086-1) (accessed on 13 November 2025) [60]. These public databases are all recognized as authoritative sources in the field. Detailed descriptions of the acquisition and use of these data are provided in the Methods section of our manuscript. All derivative data generated during our analysis are included within the manuscript and its Supplementary Materials.

## Supplementary Materials

The following supporting information can be downloaded at: https://www.mdpi.com/article/10.3390/cimb48030269/s1.
