# Supplementary material for: MVGAE: A Multi-View Graph Auto-Encoder Model for Drug Prediction of Non-Small Cell Lung Cancer Based on Synthetic Lethality
Source: Curr Issues Mol Biol. 2026 Mar 3;48(3):269. doi: 10.3390/cimb48030269 (PMC13025638; doi:10.3390/cimb48030269)
Supplement: Supplementary file 1 [file cimb-48-00269-s001.zip › cimb-4168851-supplementary.pdf]

Table S1. The parameter values involved in Section 2.5 are specified as follows.

Parameter	Value
α	0.633
β	0.992
γ	0.008
w_D	0.044
w_B	0.751
w_C	0.105
w_R	0.100

Table S2. Gene AR and drug PI-103.

**1GS4 and PI-103**						
**Hydrophobic Interactions**						
**Index**	**Residue**	**AA**	**Distance**	**Ligand Atom**	**Protein Atom**	
1	715A	VAL	3.97	20	442	
2	718A	TRP	3.54	20	474	
**Salt Bridges**						
**Index**	**Residue**	**AA**	**Distance**	**Protein positive?**	**Ligand Group**	**Ligand Atoms**
1	681A	GLU	4.58	×	Tertamine	1
2	681A	GLU	4.45	×	Tertamine	2

Table S3. Gene ATR and drug PI-103.

**5YZ0 and PI-103**					
**Hydrophobic Interactions**					
**Index**	**Residue**	**AA**	**Distance**	**Ligand Atom**	**Protein Atom**
1	555B	GLN	3.74	20	21596
2	560B	GLU	3.92	20	21626

Table S4. Gene CFLAR and drug PAZOPANIB.

**3H11 and PAZOPANIB**									
**Hydrogen Bonds**									
**Index**	**Residue**	**AA**	**Distance H-A**	**Distance D-A**	**Donor Angle**	**Protein donor?**	**Side chain**	**Donor Atom**	**Acceptor Atom**
1	374B	GLN	2.57	3.55	160.78	√	×	3557[N3+]	5[N3]
2	393B	ASN	3.13	3.58	108.02	√	√	3745[Nam]	4[Nox]
**Salt Bridges**									
**Index**	**Residue**	**AA**	**Distance**	**Protein positive?**	**Ligand Group**	**Ligand Atoms**			
1	398A	GLU	3.39	×	Tertamine	4			

Table S5. Gene CFLAR and drug TANDUTINIB.

3H11 and TANDUTINIB									
**Hydrogen Bonds**									
**Index**	**Residue**	**AA**	**Distance H-A**	**Distance D-A**	**Donor Angle**	**Protein donor?**	**Side chain**	**Donor Atom**	**Acceptor Atom**
1	423B	ASP	3.53	3.91	107.32	√	√	4062[O3]	40[O3]
2	426B	THR	2.60	3.50	156.91	√	√	4089[O3]	40[O3]
3	474A	LYS	3.20	3.79	118.42	√	√	2094[N3+]	3[N3]
**Salt Bridges**									
**Index**	**Residue**	**AA**	**Distance**	**Protein positive?**	**Ligand Group**	**Ligand Atoms**			
1	422B	ASP	4.41	×	Tertamine	6			
2	441A	ASP	4.94	×	Tertamine	4			
3	441A	ASP	4.55	×	Tertamine	2			
4	441A	ASP	5.05	×	Tertamine	1			

Table S6. Gene FBXW7 and drug PHA-793887.

2OVR and PHA-793887									
**Hydrogen Bonds**									
**Index**	**Residue**	**AA**	**Distance H-A**	**Distance D-A**	**Donor Angle**	**Protein donor?**	**Side chain**	**Donor Atom**	**Acceptor Atom**
1	2467B	MET	2.13	3.13	166.35	√	×	3383[Nam]	25[O3]
2	2507B	VAL	2.93	3.85	150.23	√	×	3774[Nam]	3[N3+]
3	2507B	VAL	2.85	3.39	115.35	×	×	3[N3+]	3777[O2]
4	2508B	GLN	3.18	3.75	116.15	√	√	3789[Nam]	26[O3]

Table S7. Gene FBXW7 and drug ALVOCIDIB.

2OVR and ALVOCIDIB									
**Hydrogen Bonds**									
**Index**	**Residue**	**AA**	**Distance H-A**	**Distance D-A**	**Donor Angle**	**Protein donor?**	**Side chain**	**Donor Atom**	**Acceptor Atom**
1	2334B	LEU	2.55	3.10	118.31	×	×	25[O3]	2081[O2]
2	2335B	HIS	2.26	3.25	162.63	√	√	2094[Npl]	23[O3]
3	2336B	ILE	3.13	3.79	123.96	√	×	2100[Nam]	26[O3]
4	2336B	ILE	3.98	4.01	108.93	×	×	23[O3]	2103[O2]
5	2353B	SER	2.21	3.06	148.23	√	√	2266[O3]	27[O3]

Table S8. Gene FBXW7 and drug DINACICLIB.

2OVR and DINACICLIB									
**Hydrogen Bonds**									
**Index**	**Residue**	**AA**	**Distance H-A**	**Distance D-A**	**Donor Angle**	**Protein donor?**	**Side chain**	**Donor Atom**	**Acceptor Atom**
1	2336B	ILE	2.81	3.59	134.21	√	×	2100[Nam]	6[N3]
2	2353B	SER	2.38	3.16	138.52	√	√	2266[O3]	29[O3]
**Salt Bridges**									
**Index**	**Residue**	**AA**	**Distance**	**Protein positive?**	**Ligand Group**	**Ligand Atoms**			
1	1156A	GLU	4.81	×	Tertamine	5			
**π-Cation Interactions**									
**Index**	**Residue**	**AA**	**Distance**	**Offset**	**Protein charged?**	**Ligand Group**	**Ligand Atoms**		
1	2348B	HIS	3.65	0.83	×	tertamine	5		

Table S9. Gene IL6ST and drug REGORAFENIB.

3L5I and REGORAFENIB									
**Hydrogen Bonds**									
**Index**	**Residue**	**AA**	**Distance H-A**	**Distance D-A**	**Donor Angle**	**Protein donor?**	**Side chain**	**Donor Atom**	**Acceptor Atom**
1	312A	TYR	3.23	3.74	126.65	×	√	4[Nox]	152[O3]

Table S10. Gene JUN and drug PHA-793887.

6Y3V and PHA-793887									
**Hydrophobic Interactions**									
**Index**	**Residue**	**AA**	**Distance**	**Ligand Atom**	**Protein Atom**				
1	119A	PHE	3.99	13	1184				
2	168A	ILE	3.66	13	1687				
**Hydrogen Bonds**									
**Index**	**Residue**	**AA**	**Distance H-A**	**Distance D-A**	**Donor Angle**	**Protein donor?**	**Side chain**	**Donor Atom**	**Acceptor Atom**
1	42A	ASN	2.63	3.32	127.07	×	√	1[Nox]	469[O2]
2	45A	SER	2.95	3.68	135.14	√	√	496[O3]	5[N3]
3	49A	LYS	3.20	3.93	128.90	√	√	535[N3+]	5[N3]
4	122A	LYS	2.48	3.30	136.34	√	√	1221[N3+]	26[O3]
5	167A	PRO	2.39	3.36	169.06	×	×	3[N3+]	1678[O2]

Table S11. Gene RAD50 and drug BORTEZOMIB.

5GOX and BORTEZOMIB									
**Hydrophobic Interactions**									
**Index**	**Residue**	**AA**	**Distance**	**Ligand Atom**	**Protein Atom**				
1	659B	LEU	3.66	27	2676				
2	664A	ALA	3.75	22	849				
3	665A	VAL	3.58	19	857				
4	666B	TYR	3.69	16	2726				
5	668A	GLN	3.84	20	885				
**Hydrogen Bonds**									
**Index**	**Residue**	**AA**	**Distance H-A**	**Distance D-A**	**Donor Angle**	**Protein donor?**	**Side chain**	**Donor Atom**	**Acceptor Atom**
1	666B	TYR	2.13	3.13	171.91	×	√	8[Nam]	2729[O3]
2	666B	TYR	2.48	3.02	115.62	√	√	2729[O3]	2[Nar]
3	672A	GLN	2.33	3.29	156.49	√	√	930[Nam]	5[Nar]
**π-Cation Interactions**									
**Index**	**Residue**	**AA**	**Distance**	**Offset**	**Protein charged?**	**Ligand Group**	**Ligand Atoms**		
1	704B	LYS	5.22	1.28	√	Aromatic	1,2,4,5,7,10		

Table S12. Gene TSC1 and drug PHA-793887.

4Z6Y and PHA-793887									
**Hydrophobic Interactions**									
**Index**	**Residue**	**AA**	**Distance**	**Ligand Atom**	**Protein Atom**				
1	944H	ALA	3.91	13	12322				
**Hydrogen Bonds**									
**Index**	**Residue**	**AA**	**Distance H-A**	**Distance D-A**	**Donor Angle**	**Protein donor?**	**Side chain**	**Donor Atom**	**Acceptor Atom**
1	944D	ALA	2.37	3.35	179.03	×	×	3[N3+]	5940[O2]
2	947D	ARG	2.90	3.80	148.15	√	√	5971[Ng+]	3[N3+]
**Salt Bridges**									
**Index**	**Residue**	**AA**	**Distance**	**Protein positive?**	**Ligand Group**	**Ligand Atoms**	**Index**		
1	949H	GLU	5.46	×	Tertamine	5	1		

Table S13. Gene TTN and drug BORTEZOMIB.

8OMW and BORTEZOMIB					
**Hydrophobic Interactions**					
**Index**	**Residue**	**AA**	**Distance**	**Ligand Atom**	**Protein Atom**
1	7A	PRO	3.75	25	66
2	28A	PRO	3.94	21	258
3	29A	LEU	3.77	19	265

4	29A	LEU	3.65	16	267
5	30A	ASP	3.66	22	274

Hydrogen Bonds									
**Index**	**Residue**	**AA**	**Distance H-A**	**Distance D-A**	**Donor Angle**	**Protein donor?**	**Side chain**	**Donor Atom**	**Acceptor Atom**
1	7A	PRO	2.68	3.66	165.64	×	×	11[Nam]	64[O2]
2	9A	GLY	3.50	3.91	108.59	×	×	9[O3]	78[O2]
3	26A	THR	2.65	3.33	138.85	√	√	244[O3]	5[Nar]
4	29A	LEU	3.22	3.76	122.63	√	×	261[Nam]	3[O2]
5	97A	GLU	2.31	3.07	147.88	√	×	905[Nam]	9[O3]
6	97A	GLU	2.33	3.22	156.41	√	√	912[O3]	12[O3]

Table S14. Summary of Identified Driver Genes, SL Partners, and Candidate Drugs

Driver Gene	Source (Score)	SL Partner	Source (Score)	Candidate Drug	Source (Score)
CDK1 [93]	NIAPU; MCS (-2.638); Topology Score (1.192)	RAD50	SLMGAE (0.173); Multi-omics (4.555)	BORTEZOMIB	MVGAE (0.061); oncoPredict(Cors = -0.480, $p = 1.042 \times 10^{-61}$)
		TTN	SLMGAE (0.179); Multi-omics (4.084)	BORTEZOMIB	MVGAE (0.025); oncoPredict (Cors = -0.504, $p = 6.200 \times 10^{-69}$)
CDC20 [93]	NIAPU; MCS (-2.241); Topology Score (1.116)	TSC1	SLMGAE (0.267); Multi-omics (5.120)	PHA-793887	MVGAE (0.045); oncoPredict (Cors = -0.349, $p = 1.317 \times 10^{-31}$)
		IL6ST	SLMGAE (0.225); Multi-omics(5.002)	REGORAFENIB	MVGAE (0.079); oncoPredict (Cors = -0.561, $p=1.988 \times 10^{-88}$)
PCNA	NIAPU; MCS(-3.134); Topology Score(0.864)	CFLAR	SLMGAE (0.144); Multi-omics(4.073)	PAZOPANIB	MVGAE (0.318); oncoPredict (Cors = -0.285, $p = 3.627 \times 10^{-21}$)
				TANDUTINIB	MVGAE (0.162); oncoPredict (Cors = -0.335, $p = 4.890 \times 10^{-29}$)
		JUN	SLMGAE (0.181); Network score (3.130)	PHA-793887	MVGAE (0.057); oncoPredict (Cors = -0.516, $p = 9.841 \times 10^{-73}$)

Driver Gene	Source (Score)	SL Partner	Source (Score)	Candidate Drug	Source (Score)
CHEK1 [94]	NIAPU; MCS(-1.824); Topology Score(1.491)	TTN	SLMGAE (0.337); Network score (3.148)	BORTEZOMIB	MVGAE (0.025); oncoPredict (Cors = -0.504, $p = 6.200 \times 10^{-69}$)
		CFLAR	SLMGAE (0.098); Network score (4.075)	PAZOPANIB	MVGAE (0.318); oncoPredict (Cors = -0.285, $p = 3.627 \times 10^{-21}$)
				TANDUTINIB	MVGAE (0.162); oncoPredict (Cors = -0.335, $p = 4.890 \times 10^{-29}$)
DDB1	NIAPU; MCS(-2.180); Topology Score(0.721)	RAD50	SLMGAE (0.113); Network score (3.912)	BORTEZOMIB	MVGAE (0.061); oncoPredict(Cors = -0.480, $p = 1.042 \times 10^{-61}$)
		ATR	SLMGAE (0.237); Network score (1.463)	PI-103	MVGAE (0.068); oncoPredict (Cors = -0.556, $p = 1.454 \times 10^{-86}$)
RAD51 [95]	NIAPU; MCS(-1.324); Topology Score(0.952)	ATR [91]	SLMGAE (0.360); Network score (0.917)	PI-103	MVGAE (0.068); oncoPredict (Cors = -0.556, $p = 1.454 \times 10^{-86}$)
		FBXW7	SLMGAE (0.227); Network score (2.620)	PHA-793887	MVGAE (0.009); oncoPredict (Cors = -0.547, $p = 3.510 \times 10^{-83}$)
				ALVOCIDIB	MVGAE (0.050); oncoPredict (Cors = -0.601, $p = 9.001 \times 10^{-105}$)
				DINACICLIB	MVGAE (0.103); oncoPredict (Cors = -0.547, $p = 5.798 \times 10^{-58}$)
CYCS	NIAPU; MCS(-1.091); Topology Score(0.725)	JUN	SLMGAE (0.264); Network score (1.795)	PHA-793887	MVGAE (0.057) ; oncoPredict (Cors = -0.516, $p = 9.841 \times 10^{-73}$)
		AR	SLMGAE (0.256); Network score (1.479)	PI-103	MVGAE (0.080); oncoPredict (Cors = -0.310, $p = 7.270 \times 10^{-25}$)

Table 15. Stability Comparison of PR and ROC Curves Before and After Incorporating GO_MF

Metric	SLMGAE	SLMGAE+MF	Improvement
**ROC Curve**			
Mean Std Dev	0.0187	0.0179	+4.36%
Max Std Dev	0.1451	0.0622	+57.13%
**PR Curve**			
Mean Std Dev	0.0126	0.0099	+21.27%
Max Std Dev	0.0945	0.0887	+6.54%
